# Analysis of seminal plasma from brown bear (*Ursus arctos*) during the breeding season: Its relationship with testosterone levels

**DOI:** 10.1371/journal.pone.0181776

**Published:** 2017-08-03

**Authors:** L. Anel-López, C. Ortega-Ferrusola, C. Martínez-Rodríguez, M. Álvarez, S. Borragán, C. Chamorro, F. J. Peña, L. Anel, P. de Paz

**Affiliations:** 1 Animal Reproduction and Obstetrics, University of León, Spain; 2 ITRA-ULE, INDEGSAL, University of León, León, Spain; 3 Molecular Biology (Cell Biology), University of León, León, Spain; 4 Cabárceno Park, Cantabria, Spain; 5 Veterinary Anatomy, University of León, León, Spain; 6 Laboratory of Equine Reproduction and Equine Spermatology, Veterinary Teaching Hospital, University of Extremadura, Cáceres, Spain; Senckenberg am Meer Deutsches Zentrum fur Marine Biodiversitatsforschung, GERMANY

## Abstract

Seminal plasma (SP) plays an important role in the motility, viability and maintenance of the fertilizing capacity of mammalian spermatozoa. This study is the first on brown bear (*Ursus arctos*) SP components, and has two main objectives: 1) to define the SP composition in bear ejaculate and 2) to identify variations in SP composition in relation to high and low levels of testosterone in serum during the breeding season. Forty-eight sperm samples from 30 sexually mature male brown bears (*Ursus arctos*) were obtained by electroejaculation, and their serum testosterone levels were assessed to sort the animals into 2 groups (high and low testosterone levels, threshold 5 ng/dl). The biochemical and protein compositions of the SP samples were assessed, and sperm motility was analyzed. We found that lactate dehydrogenase was significantly higher in the low-serum-testosterone samples, while concentrations of lipase and Mg^+^ values were significantly higher in the high-serum-testosterone samples. In contrast, sperm motility did not significantly differ (P>0.05) between the testosterone level groups (total motility: 74.42.8% in the high-level group vs. 77.1±4.7% in the low-level group). A reference digital model was constructed since there is no information for this wild species. To do this, all gel images were added in a binary multidimensional image and thirty-three spots were identified as the most-repeated spots. An analysis of these proteins was done by qualitative equivalency (isoelectric point and molecular weight) with published data for a bull. SP protein composition was compared between bears with high and low serum testosterone, and three proteins (binder of sperm and two enzymes not identified in the reference bull) showed significant (P<0.05) quantitative differences. We conclude that male bears with high or low serum testosterone levels differs only in some properties of their SP, differences in enzyme LDIP2, energy source LACT2, one protein (similar to BSP1) and Mg ion were identified between these two groups. These data may inform the application of SP to improve bear semen extenders.

## 1. Introduction

According to mitochondrial DNA studies [1], the Cantabrian brown bear (*Ursus arctos cantabricus*) is the only existing pure brown bear population on the Iberian Peninsula. Habitat loss and population fragmentation have resulted in a significant reduction in the number of bears, so this species is currently considered to be at risk of extinction (Real Decreto 439/1990, Regulation of the National Catalogue of Endangered Species). Artificial insemination is one of the most valuable tools in *ex situ* reproduction programs for endangered animals, so sperm cryopreservation is very important to the creation of a genetic resource bank for the brown bear [2–4]. Genetic variability could be restored to the endangered wild population by storing sperm samples in genetic banks.

In recent years, intensive research has been done to describe the composition of seminal plasma (SP) and to characterize its proteins [5,6]. SP is a complex fluid that contains different components, such as proteins, enzymes, macro- and microelements, lipids and nutrients, and it plays an important role in spermatozoa motility, viability and the maintenance of fertilizing capacity [5,6]. Furthermore, some authors have observed that the proteins contained in SP influence the female genital tract and ovum fertilization [5,7,8]. Various SP components (enzymes, sources of energy, minerals) can be measured to assess accessory sex organ function and their relation to semen quality. The impact of calcium, magnesium, and zinc on sperm motility, count and morphology were determined in men; only weak correlations were demonstrated and the determination of these elements not discriminate males on the basis of fertility [9]. Analysis of various enzyme activities and its influence on sperm function has been reported, among others alkaline phosphatase and lactate dehydrogenase [10], lipase [11] and glutamic oxaloacetic transaminase [12].

Different authors have been studying the effect of adding SP (the whole fraction or specific proteins) to the sperm dilution media (extender) on the maintenance of sperm quality in chilled [13], cryopreserved [14] and sex-sorted sperm [7]. While some studies have demonstrated that SP addition decreases the fertilizing capacity of the spermatozoa [15–17], other authors have found that it leads to an increase in viability [18], fertilizing capacity [19–21] and cold-shock resistance [13,14,22,23]. However, the effect of SP on sperm quality is very variable and depends on seasonal variations in plasma seminal composition, among other factors [24].

Until now, the composition of brown bear SP has been unknown. In addition to individual variations in SP composition between ejaculate portions [25] or even among animal breeds [26], respect to reproductive season have been described in several species (boar [27], goat [28] and ram [29,30]). SP is secreted from various structures (prostate gland and accessory sex glands) in the male reproductive system [31,32] whose activity depends on testosterone levels [33,34]. The brown bear is a species that exhibits strong breeding seasonality, so we can hypothesize about the relationships between testosterone levels during the breeding season and changes in SP biochemical and protein composition and sperm motility.

This is the first study of various components of SP in the brown bear, and it has two main objectives: 1) to define the SP composition in bear ejaculate and 2) to identify variations in SP composition in relation to high and low levels of testosterone in serum during the breeding season and their effect on sperm quality.

## 2. Materials and methods

### Bear management and sperm collection

Animal handling and electroejaculation were performed in accordance with the Spanish Animal Protection Regulation RD53/2013, which conforms to European Union Regulation 2010/63/UE. All experiments were performed after obtaining approval from the Ethical Committee for Experimentation with Animals of León University, Spain (03-02/2010).

Forty-eight sperm samples from 30 sexually mature male brown bears were obtained by electroejaculation during the breeding season. Animals were housed under a half-freedom in Cabarceno Park (Cantabria, Spain) and fed ad libitum a diet based on chicken meat, bread and fruits. The males were captured by tele-anesthesia using darts containing a mixture of Zolazepam-tiletamine (7 mg/kg, Zoletil100®; Virbac, Carros, France) and ketamine (2 mg/ kg, Imalgene 1000®; Rhone-129 Mérieux, Lyon, France). After capture, the bears were immobilized. During the anesthesia, pulse, peripheral oxygen saturation and breathing of the bears were monitored (Ohmeda 3800, GE Healthcare, Finland). Prior to electroejaculation, the genital region was cleaned; the prepuce was shaved; the penis was washed with sterile physiological saline; and the bladder was catheterized to avoid urine contamination in semen samples. Electroejaculations were carried out with a PT Electronics1 electroejaculator (PT Electronics, Boring, OR, USA), after removing the feces from the rectum, using a 320-mm x 26-mm transrectal probe; electrical stimuli were applied until ejaculation (1–8 V and 250–300 mA, on average). The electroejaculation process required an average of 10 min to complete, and two electroejaculation sessions were performed for each bear with a 15-30-min interval between them. To prevent urine contamination or a low cell concentration, the ejaculates were collected as isolated fractions in 15-mL graduated glass tubes. Immediately after collection, the volume and concentration of each ejaculate were assessed; osmolality was measured using an Osmomat-030 cryoscopic osmometer (Gonotec, Berlin, Germany); and the pH value was determined (CG 837 pH meter; Schott Instruments, Main, Germany). Urospermia was evaluated by means of a rapid urea test (urea test strips, DiaSys Ecoline®GmbH, Holzheim, Germany) and urine-contaminated samples (>80 mg urea/dL) were rejected.

Samples were assessed for motility and concentration just after collection. First, a warmed Makler counting chamber was loaded with 5 μL of sample to evaluate concentration and the analysis was carried out using a CASA (computer-assisted sperm analysis) system consisting of an optical phase-contrast microscope (Nikon Labophot-2, Tokyo, Japan; fitted with negative phase-contrast objectives and a warming stage at 37°C), a Basler A312fc camera (Basler, Germany), and a PC with Sperm Class Analyzer software (ISAS v. 1.2; Proiser, Valencia, Spain); the magnification was 100X. Second, an aliquot of semen was diluted with a TES–Tris–Fructose extender to 25x10^6^ cell/ml and motility was analyzed by CASA. At least five fields were acquired per sample at a rate of 25 images per second, which recorded a total of 200 motile sperm. The following parameters were used: total motility (%TM) and progressive motility (%PM). Image sequences were saved and later analyzed using the editing facilities provided by ISAS. Non-spermatozoa events were removed and settings were adjusted in each case to assure correct tracking analysis. Sperm were considered motile when VCL > 10 μm/sec and progressive if VCL >25 and straightness (STR) >80% [35].

### Seminal plasma analysis

Each semen sample was centrifuged at 600 x g for 6 min to obtain a sperm pellet and a liquid fraction (seminal plasma). The SP was collected by micropipette and stored in 2-ml cryotubes at -80°C until use. Prior to the SP extraction, the samples were again centrifuged at 18000 g to obtain a sample free of cells. Sperm pellet was diluted to 25x10^6^ with a TES–Tris–Fructose extender and cryopreserved as described in a previous study [3].

A COBAS Integra 400 analyzer (Roche Diagnostic, Mannheim, Germany) was used to measure the following parameters: ALP (alkaline phosphatase; U/L), GOT (glutamic oxaloacetic transaminase; U/L), LDIP2 (lactate dehydrogenase; U/L), LIPC (colorimetric lipase; U/L), GLUC3 (glucose; mg/dL), LACT2 (lactate; mmol/L), Mg (mg/dL), Ca (mmol/L), POSH2 (phosphate; mg/dL), and CHOL2 (cholesterol; mmol/L). COBAS calibration was done with C.fas calibration serum (Roche Diagnostic, Mannheim, Germany), and Precinorm U (Roche Diagnostic, Mannheim, Germany) and Precipath U (Roche Diagnostic, Mannheim, Germany) were used as normal and pathologic controls, respectively.

Zn (μg/dL) was evaluated using a BA400 analyzer (Biosystem, Barcelona, España). Analyzer calibration was done with R3 CAL ZINC liquid (BEN, Milano, Italy) and BROMO-PAPS (Biosystem, Barcelona, España) was used as reagent.

### Testosterone analysis and classification

**Fig 1:**
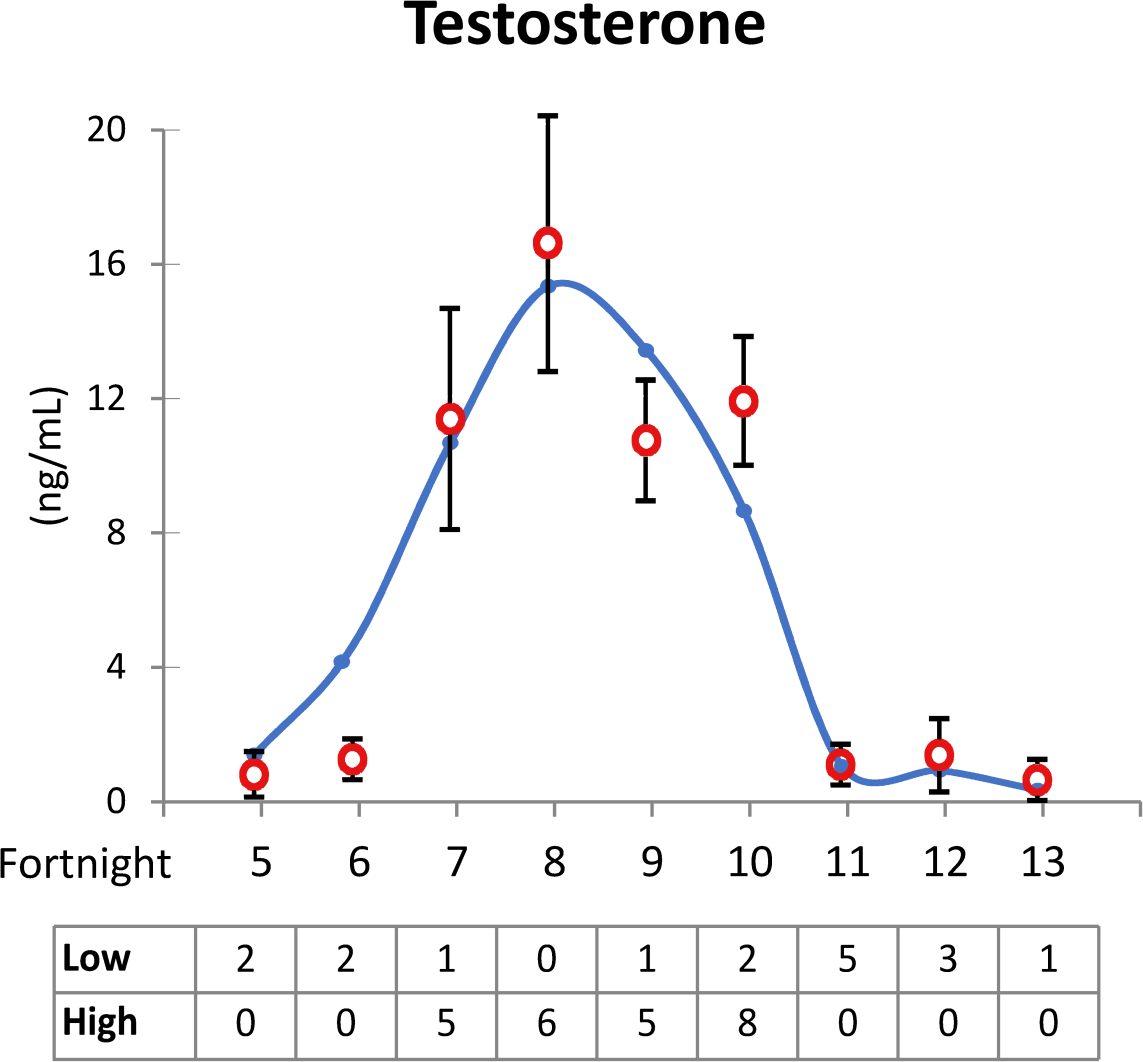
Distribution of brown bear testosterone levels between fortnights 5 and 13. The curve shows the distribution of the testosterone levels among the whole bear population (n = 122) and the open points shows data of bears included in the present study (mean ± S.E.M., n = 41). The values below represent the number of bears with high or low testosterone levels included in this study for that fortnight.

Blood (10 ml) was collected from the cephalic vein within 10:00 and 11:30 h. Blood sample was left standing for 5 h to form a coagulum and then centrifuged at 3500 g for 15 min. Serum sample was immediately separated and stored in vials at -20°C until hormone assay. Serum testosterone levels were assessed using an IMMULITE^®^ 1000 Total Testosterone System commercial kit (Siemens, Glyn Rhonwy, United Kingdom), and male bears were sorted into groups with either high or low testosterone levels using 5 ng/dL as a threshold. The brown bear is a species with strong reproductive seasonality, so males with the highest reproductive activity were located between fortnights 7 and 10 (Fig 1), which is the period with highest testosterone levels (above 5 ng/dL). Fig 1 is a curve obtained from historical database (122 samples).

### Bear seminal plasma 2D electrophoresis

A Quick Start™ Bradford Protein Assay (Bio-Rad Laboratories, Madrid, Spain) was used to quantify the protein concentrations in the samples.

#### Determination of SP protein concentrations

Eight volumes of cold acetone (-20°C) were added to the SP samples, and this mixture was briefly vortexed. Then, a volume of 100% TCA was added; the mixture was vortexed again; and the tube was incubated for a minimum of 3 h at -20°C. Afterwards, the tube was centrifuged at 16000–18000 x g for 15 min at 4°C; the supernatant was discarded; the pellet was resuspended in cold acetone (-20°C); the tube was left for 10 min at -20°C; and the tube was centrifuged again under the same conditions as before to obtain the protein pellet. To solubilize the pellet, the required amount of UTCh buffer (urea 7 M, thiourea 2 M, 4% CHAPS) supplemented with 50 mM of dithiothreitol (DTT) was used to attain a final protein concentration of 20 mg/mL in the sample.

#### Isoelectric focusing (IEF)

First dimension. Proteins were separated using 7-cm 12% polyacrylamide strips (General Electrics, Uppsala, Sweden) with pH values from 4 to 7, and 125 μl of a solution containing UTCh buffer plus 50 mM DTT, 0.2% Bio-Lyte ampholyte (Bio-Rad, USA), 0.0002% bromophenol blue and 100 μg of proteins were prepared for strip rehydration. Once the strips were rehydrated, the IEF was run in 4 steps at constant current (2 mA): 1) quick ramp for 30 min at 250 V, 2) linear ramp for 1 h at a maximum of 1000 V, 3) linear ramp for 2 h at a maximum of 4500 V, 4) linear ramp until 8000 V was reached. The strips were then maintained at 500 V until they were removed from the machine and stored at -20°C.

Second dimension. The strips were first equilibrated in an equilibration buffer containing 6 M urea, 0.05 M Tris-HCl at pH 8.8, 20% glycerol, 2% sodium dodecyl sulfate (SDS) and 2% DTT, and then again equilibrated in the same equilibration buffer plus 2.5% iodoacetamide. The second dimension was run in a 12% polyacrylamide gel using a Miniprotean II system (Bio-Rad, USA) at 150 V. We used the SDS-PAGE Molecular Weight Standards, Broad Range (Bio-Rad, USA) as a molecular weight ladder.

### Gel analysis

The Coomasie colloidal blue silver technique was used to stain the gels as described by Candiano et al.[36]. Once stained, the gels were scanned with a densitometer (Bio-Rad GS-800 Calibrated Densitometer) for subsequent analysis.

#### Semi-quantitative analysis

**Fig 2:**
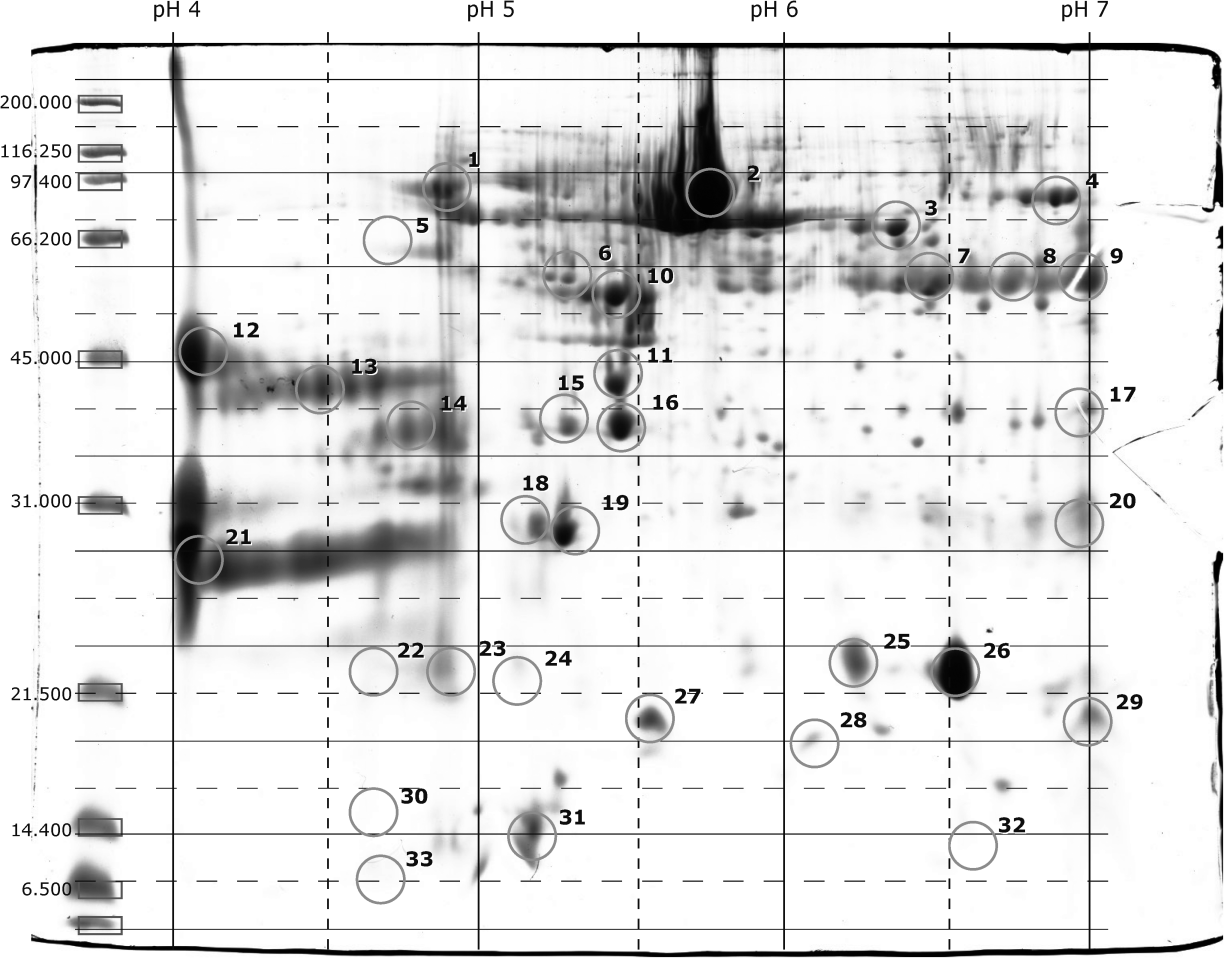
Spot identification. All the gels were overlaid for comparison. Those spots with a repeatability greater than 40% and a presence higher than 2 were considered in the computer-assisted assessment.

All photographs of the gels were digitally modified to produces images with spots over a transparent background and then "digitally added" to a binary multidimensional image that was used as a reference for stacking the isoelectric point (pI) and molecular weight (MW). This global image showed all of the protein spots that can be found in the gels; a total of 33 spots were identified and labeled with a number and their corresponding pI value and molecular weight. The gels were reanalyzed by checking the presence of each of the 33 spots, and a table was constructed in which each spot was assigned a code from 1 to 5 (1 indicated that the spot was present in less than 20% of the gels, and 5 indicated presence in 100% of the samples; each increase in the code represented a 20% increase in spot intensity). After classification, we selected those spots with values from 3 to 5 and created a model gel with the highest number of representative spots (greater than 40% repeatability, Fig 2).

#### Automatic quantitative analysis

Prior to the automatic quantitative analysis, the model gel was compared with a known buffalo (*Bos javanicus)* model, described by Sarsaifi et al. [37], for the region between pI (5–7) and MW (13–75 kDa). The spots in the two models with highly correlated molecular weights and isoelectric points were quantitatively analyzed and then compared between the high-testosterone group and the low-testosterone group using ImageMaster^TM^ 2D Platinum Software 5.0 (GE Healthcare life sciences, Amersham, Barcelona, Spain).

A preliminary analysis evaluated the quality of gels for quantitative analysis and discarded those that for technical reasons (distortion, low definition) could skew the analysis. A group of 10 gels from each testosterone level were finally analyzed and a manual editing was performed to check for cracks and other strange signals. For each gel, all spots were identified and a reference gel was defined according to the total number of detected spots. All gels were studied by overlapping in transparency mode with the reference gel, the deviation vectors were evaluated and finally the spots were identified. In each gel, the landmarks and the reference points of pI and MW are placed, the matching between classes was calculated using a Student's t test, and the volume and area were registered for the spots of interest [38].

### Statistical analysis

Data were tested for normality (UNIVARIATE procedure) and arcsin square-root transformation was used to normalize the data before analysis when was necessary. Data were analyzed using the GLM procedures of program SAS™ v.9.1. (SAS Institute Inc., Cary, NC, USA) and a variance homogeneity test was used (Levene’s test). Mean comparisons were obtained by Tukey contrasts, and the results are presented as the mean±SEM (standard error of the mean) using significance level of *P* < 0.05.

## 3. Results

Fresh semen samples showed an average volume of 2.55 ±0.23 and 2.35 ±0.24 ml, and a sperm concentration of 206.88 ± 49.2 × 106 and 163.68 ± 30.1 × 106 sperm ml^−1^, for brown bear with high and low testosterone level respectively. Males with high testosterone levels showed a semen with pH 8.3 (±0.06) and osmolality 292.1 (±7.1) versus pH 8.23 (±0.07) and osmolality 294.2 (±4.9) in males with low levels.

### Biochemical analysis

SP biochemical profiles were compared between males with high or low serum testosterone levels (Table 1). Among the analyzed parameters, we found that LDIP2 showed significantly higher values in the low-testosterone samples, while LACT2 and Mg showed significantly higher values in the high-testosterone samples.

**Table 1:** Biochemical evaluation of bear seminal plasma (concentration, mean ± S.E.M.) classified according to the high or low levels of serum testosterone.

	TESTOSTERONE		
	High (n = 24)	Low (n = 17)	
**ALP** (U/L)	1096.7±239.9	1192.2±196.3	
**GOT** (U/L)	89.4±13.8	90.9±14.9	
**LDIP2** (U/L)	98.9±22.9	219.2±59.3	*
**LIPC** (U/L)	5.3±0.5	5.8±0.6	
**GLUC3** (mg/dL)	13.1±2.1	16.5±3.5	
**LACT2 (**mmol/L)	5.1±0.4	3.8±0.3	*
**PHOS2** (mg/dL)	4.4±0.8	6.2±1.9	
**CHOL2 (**mmol/L)	0.1±0.01	0.1±0.02	
**Ca (**mmol/L)	0.4±0.1	0.5±0.1	
**Mg** (mg/dL)	2.6±0.4	1.3±0.2	*
**Zn** (mg/dL)**	28.7±4.1	26.3±2.9	

ALP (alkaline phosphatase), GOT (glutamic oxaloacetic transaminase), LDIP2 (lactate dehydrogenase), LIPC (colorimetric lipase), GLUC3 (glucose), LACT2 (lactate), POSH2 (phosphate), CHOL2 (cholesterol), Mg (magnesium), Ca (calcium), Zn (zinc).

* indicates significant differences (P<0.05) between high and low testosterone groups in that parameter.

** n = 8.

### Sperm motility assessment

Sperm motility did not differ (P>0.05) between the high- and low-testosterone groups in total motility (High_Test: 74.4 ± 2.8% vs. Low_Test: 77.1 ± 4.3%) or progressive motility (High_Test: 43.6 ± 3.3% vs. Low_Test: 45.1 ± 3.6%).

### Proteomic analysis

The comparison of the bear model gel with the known pattern described by Sarsaifi et al. [29] in a bull model showed 16 spots with similar pI and MW data. These spots in the bear gels were characterized in the bull gel as 2 serum albumins, 1 nucleobinding, 3 phospholipases, 1 ALB protein, 3 clusterins, 2 binding of sperm (BSP) and 2 spermadhesins; 2 spots were unknown proteins (Table 2). The sixteen spots from the bear gels that were coincident with those of the bull gels were quantified in bears with low and high levels of testosterone by automatic analysis. We compared these SP proteomic profiles and found a significant difference in the relative presence of protein in 3 spots (Table 3). The protein identified with pI 5.2 and MW 30 kDa (BSP1) showed significantly higher values (P<0.05) in low-testosterone than high-testosterone samples. Conversely, proteins of spots C16 and C18 (not identified) showed higher values in the samples with high-testosterone levels.

**Table 2:** Spots from 2D electrophoresis of bear seminal plasma gels equivalent to those from bull gels [37] according to molecular weight and isoelectric point.

Bear Spot*	MW (kDa)	pI	Bull Spot**	MW (kDa)	pI
C2	80	5.6	Serum Albumin	70‐75	5.8
C3	75	6	Serum Albumin	70‐75	5.8
C5	66	4.8	Nucleobindin-1 (49)	70‐75	4.8‐5.2
C6	60	5.4	Phospholipase A2	50‐55	6
C7	60	6.1	ALB Protein	68‐70	5.5‐6
C10	55	5.3	Phospholipase A2	50‐55	6
C11	54	5.4	Phospholipase A2	50‐55	6
C12	45	4.1	37 Clusterin	37‐45	4‐5.2
C13	43	4.3	37 Clusterin	37‐45	4‐5.2
C14	38	4.6	37 Clusterin	37‐45	4‐5.2
C16	40	5.3	Not Identified	37‐40	5.3‐5.5
C18	30	5.1	Not Identified	25‐30	5.2‐5.5
C19	30	5.2	BSP1	25‐30	5.3‐5.6
C21	28	4	BSP5	25‐30	3.0‐4.0
C26	22	6.2	Spermadhesin	13‐14	5.5‐5.8
C32	12	6.4	Spermadhesin	13‐14	5.5‐5.8

Spots from bear gels and bull gels (proteins) identified by molecular weight (kDa) and isoelectric point.

* Generic name according to order of appearance in the gel.

** Abbreviations: BSP: binder of sperm; ALB: albumin.

**Table 3:** Automatic quantitative analysis. Means volume percentages for the spots from males with high and low levels of testosterone.

Bear Spot	MW (kDa)	pI	Bull Protein	MW (kDa)	pI	High Testosterone	Low Testosterone	
C2	80	5.6	Serum Albumin	70‐75	5.8	0.111 (±0.026)	0.106 (±0.020)	
C3	75	6	Serum Albumin	70‐75	5.8	0.330 (±0.053)	0.233 (±0.037)	
C6	60	5.4	Phospholipase A2	50‐55	6	0.081 (±0.012)	0.053 (±0.008)	
C7	60	6.1	ALB Protein	68‐70	5.5‐6	0.123 (±0.013)	0.092 (±0.009)	
C10	55	5.3	Phospholipase A2	50‐55	6	0.066 (±0.010)	0.065 (±0.000)	
C11	54	5.4	Phospholipase A2	50‐55	6	0.061 (±0.007)	0.094 (±0.017)	
C14	38	4.6	37 Clusterin	37‐45	4‐5.2	0.354 (±0.055)	0.347 (±0.070)	
C16	40	5.3	Not Identified	37‐40	5.3‐5.5	0.134 (±0.026)	0.071 (±0.021)	*
C18	30	5.1	Not Identified	25‐30	5.2‐5.5	0.224 (±0.035)	0.124 (±0.022)	*
C19	30	5.2	BSP1	25‐30	5.3‐5.6	0.042 (±0.004)	0.104 (±0.026)	*
C32	12	6.4	Spermadhesin	13‐14	5.5‐5.8	0.098 (±0.024)	0.078 (±0.016)	

(*) Indicates a significant difference (P<0.05) in the relative presence of that spot between high and low levels of testosterone.

Abbreviations: BSP: binder of sperm; ALB: albumin.

## 4. Discussion

The composition of seminal plasma and its relationship with the serum testosterone levels in brown bear during the breeding season have been assessed in this work. Our results show no statistical difference in plasma seminal osmolality and pH or sperm motility and count between low and high serum testosterone groups. A slight positive trend between serum testosterone and sperm count is reported. Semen composition and the properties of the SP differs among species as well as among individuals within species or breeds [21,22]. Several authors have described the relationship between SP and spermatozoa motility, viability and the maintenance of fertilizing capacity [5,6], as well as the influence of SP on the female genital tract and ovum fertilization [5,7,8]. In the present study, sperm motility was similar between males with high or low testosterone, thus the serum testosterone level does not present a positive correlation with sperm motility which has been demonstrated by other authors [39]; however Gonzales et al [40] also found that sperm motility were not related with serum testosterone levels in men living at high altitudes and these authors suggest that a resistance to the testosterone action could be occurring within the reproductive tract level.

In SP, different enzymes, such as alkaline phosphatase or lactate dehydrogenase, play important roles in the metabolic processes that provide energy to promote spermatozoa survival, motility and fertility potential [41,42]. In our study, the concentration of alkaline phosphatase did not differ between bears with high and low testosterone levels, but higher values of lactate dehydrogenase were found in low-testosterone samples than in those with a higher level. Lactate dehydrogenase is required to convert lactate and pyruvate, which is essential to sperm motility, capacitation and fertility [42]. These data can indicate that the males with high testosterone level, once they have reached a high level of lactate, a feedback mechanism is activated that causes a reduction of the concentration of the enzyme LDIP2.

No differences were found between high- and low-testosterone samples for glucose, cholesterol, phosphate, calcium or zinc, but magnesium and lactate showed higher values in the high-testosterone samples. Lactate and pyruvate are the most important sources of energy for stallion (*Equus ferus*) sperm motility and velocity, and they elicit a dose-dependent response [43], which supports the results of our study showing higher levels of lactate in the group of male bears with high levels of testosterone, in which spermatozoa are expected to be more metabolically active in female reproductive tract. Moreover, our results show that glucose concentration does not change between the two groups of males with different levels of testosterone and it is possible that this status is associated with versatile capacity of sperm to operate depending on the substrates available [43].

Semen quality has been associated with the mineral concentration in SP [44], but in the present study, only magnesium concentration changed in association with testosterone level although sperm motility not change between males with different plasma testosterone level. This mineral was negatively correlated with ram sperm motility [45] and Wong et al. [9] not find any correlation Mg and man sperm motility. Additionally, the addition of magnesium to boar extenders has been shown to improve sperm vitality [44]. Similar to our results, the men SP concentrations of calcium and zinc did not differ significantly between fertile and subfertile males, although both minerals have been associated with spermatogenesis and fertility [9,42]. However, Colagar et al. [39] demonstrated significantly higher seminal Zn levels in fertile men compared with infertile subjects. Other seasonal changes in the biochemical parameters of SP could indicate that these enzymes (catalase, superoxide dismutase, glutathione reductase or glutathione peroxidase) are involved in the sperm antioxidant system [46]. Glutathione reductase was previously shown to be the only enzyme that increased in the SP of rams with high testosterone levels during the breeding season, while the other enzymes that were assessed, such as catalase, superoxide dismutase or glutathione peroxidase, did not differ between individuals with high and low testosterone levels. These variations in SP enzymes were not correlated with testosterone levels in ram seminal plasma but showed a high correlation with melatonin level. A positive correlation has been observed between the motility of stallion sperm and the levels of glutamic oxaloacetic transaminase (GOT), glutamic pyruvic transaminase (GPT) and lactate dehydrogenase (LDH) [12]. GOT and GPT enzymes were determined to have an important role in the protection of spermatozoa from oxidative stress [12]; however other authors have investigated activities of GOT, GPT and ALP as markers of plasma oxidative status in rats hepatic function [47]. Our results showed no changes in the level of GOT and ALP associated with testosterone level, suggesting these markers that the oxidative status of bear SP no changes during the reproductive period. It is known that testosterone is required for sperm production and sperm maturation and that it affects sperm quality [48], but our results showed that brown bear sperm motility is not correlated with the level of testosterone. Testosterone is metabolized to estrogen by the activity of aromatase it has been suggested that estrogens positively regulate sperm motility [49]; also, a positive correlation has been found between sperm motility and the expression of aromatase ((human [50], buffalo [51] or ram [46]. Thus, these factors should be studied in the future in order to interpret the relationship between testosterone and sperm motility in bear.

The different proteins in SP have been widely studied since they are known to play several important roles in regulating processes related to fertility such as capacitation, the establishment of the oviductal sperm reservoir, the modulation of the uterine immune response and gamete interaction and cell fusion [51–54]. In our study, we identified spot C19 in the brown bear gels as the BSP1 protein described by Sarsaifi et al. [37] in a bull model based on its corresponding molecular weight and isoelectric point, and our results showed that males with low testosterone have lower levels of this protein than those with high testosterone. After ejaculation, the BSP proteins cover the sperm surface and interact with phospholipids that contain phosphorylcholine head groups. In the female genital track, the sperm will interact with high-density lipoproteins (HDLs), which allows the BSPs to join to the sperm membrane. Next, the BSPs sequester cholesterol and phospholipids and leave the sperm surface while forming a complex with HDL. This loss of lipids alters cell membrane permeability, allowing the calcium to enter and activate the phospholipase A_2_ (PLA_2_), which converts phospholipids to lysophospholipids that are known to destabilize cell membranes and negatively affect sperm quality [55]. Similarly, Manjunath et al. [56] showed that BSPs negatively affect sperm storage and suggested that the low-density fraction of the egg yolk has a very high capacity for BSP protein binding, preventing the interaction with the HDL from the female genital tract and thus preventing the deleterious effect on the sperm membrane. In this way, the lower level of spot C19 (identified as BSP1) observed in bears with higher testosterone levels could be beneficial for bear sperm banking since this protein could reduce damage to sperm quality.

This study examines, for the first time, the biochemical composition of SP from male brown bears during the breeding season. We conclude that, in general, there are no differences in the biochemical composition or protein content of the two groups of male bears with different levels of testosterone, but three proteins (one identified as BSP1 and two unidentified), one enzyme (LDIP2), one energy source (LACT2) and Mg ion differed. Our findings pave the way for further investigations focused on characterizing each spot found in this study and their possible applications to improve other artificial reproductive techniques.

## Supporting information

S1 File A file included with the name “RAW Data_Anel Lopez 2017.zip” have all the extra data from where we obtained the data presented in this work as follow: Data A; This is the global testosterone curve_data. Data B This is the testosterone level//bears 01–44. Data C; These are the seminal plasma gels//Bears 01–48. Data D; This is the biochemical data. Data E; This is the Zn level. Data F; Gels INBIOTEC assessment. Data G; Sperm basic and CASA parameters_database.
(ZIP)
