# Supplementary material for: Analysis of seminal plasma from brown bear (Ursus arctos) during the breeding season: Its relationship with testosterone levels
Source: PLoS One. 2017 Aug 3;12(8):e0181776. doi: 10.1371/journal.pone.0181776 (PMC5542667; doi:10.1371/journal.pone.0181776)
Supplement: S1 File — A file included with the name “RAW Data_Anel Lopez 2017.zip” have all the extra data from where we obtained the data presented in this work as follow: Data A; This is the global testosterone curve_data. Data B This is the testosterone level//bears 01–44. Data C; These are the seminal plasma gels//Bears 01–48. Data D; This is the biochemical data. Data E; This is the Zn level. Data F; Gels INBIOTEC assessment. Data G; Sperm basic and CASA parameters_database. (ZIP) [file pone.0181776.s001.zip › RAW Data_Anel Lopez 2017_REVISED/Data A.pdf]

Sample	Quincena	Testosterona (ng/mL)
S01	5	0,00
S02	5	1,74
S03	5	1,81
S04	5	3,59
S05	5	0,09
S06	5	0,97
S07	5	0,08
S08	5	1,43
S09	6	5,74
S10	6	5,99
S11	6	1,83
S12	6	4,00
S13	6	3,50
S14	6	11,20
S15	6	0,80
S16	6	1,89
S17	7	7,12
S18	7	4,31
S19	7	1,96
S20	7	6,16
S21	7	30,00
S22	7	12,91
S23	7	12,93
S24	7	19,28
S25	7	7,75
S26	7	12,05
S27	7	5,57
S28	7	30,00
S29	7	1,47
S30	7	20,91
S31	7	5,74
S32	7	3,18
S33	7	5,85
S34	7	2,25
S35	7	6,46
S36	8	17,47
S37	8	30,00
S38	8	9,48
S39	8	8,92
S40	8	2,30
S41	8	8,46
S42	8	9,76
S43	8	30,00
S44	8	26,75
S45	8	23,63

S46	8	26,59
S47	8	11,04
S48	8	13,09
S49	8	5,30
S50	8	0,72
S51	8	14,49
S52	9	15,31
S53	9	30,00
S54	9	2,61
S55	9	2,54
S56	9	30,00
S57	9	14,16
S58	9	6,65
S59	9	2,69
S60	9	15,79
S61	9	21,59
S62	9	11,57
S63	9	1,36
S64	9	13,80
S65	9	14,02
S66	10	6,49
S67	10	12,17
S68	10	16,11
S69	10	2,28
S70	10	6,45
S71	10	3,23
S72	10	4,13
S73	10	28,94
S74	10	1,19
S75	10	15,83
S76	10	2,23
S77	10	19,52
S78	10	0,17
S79	10	0,79
S80	10	4,82
S81	10	8,88
S82	11	1,40
S83	11	4,58
S84	11	0,38
S85	11	0,67
S86	11	0,27
S87	11	1,76
S88	11	0,00
S89	11	1,02
S90	11	0,49
S91	11	0,09

S92	11	0,18
S93	11	1,98
S94	11	0,20
S95	11	0,77
S96	11	0,07
S97	11	0,81
S98	11	0,15
S99	11	1,06
S100	12	0,09
S101	12	4,61
S102	12	3,75
S103	12	0,02
S104	12	0,74
S105	12	0,00
S106	12	0,45
S107	12	0,14
S108	12	0,72
S109	12	0,39
S110	12	0,43
S111	12	0,05
S112	12	0,00
S113	12	0,00
S114	12	0,00
S115	13	0,10
S116	13	0,38
S117	13	0,09
S118	13	0,85
S119	13	0,00
S120	13	0,07
S121	13	0,00
S122	13	0,00
