# Supplementary material for: Analysis of seminal plasma from brown bear (Ursus arctos) during the breeding season: Its relationship with testosterone levels
Source: PLoS One. 2017 Aug 3;12(8):e0181776. doi: 10.1371/journal.pone.0181776 (PMC5542667; doi:10.1371/journal.pone.0181776)
Supplement: S1 File — A file included with the name “RAW Data_Anel Lopez 2017.zip” have all the extra data from where we obtained the data presented in this work as follow: Data A; This is the global testosterone curve_data. Data B This is the testosterone level//bears 01–44. Data C; These are the seminal plasma gels//Bears 01–48. Data D; This is the biochemical data. Data E; This is the Zn level. Data F; Gels INBIOTEC assessment. Data G; Sperm basic and CASA parameters_database. (ZIP) [file pone.0181776.s001.zip › RAW Data_Anel Lopez 2017_REVISED/Data B.pdf]

Quincena	Bear	Testosterona	TESTOSTERONA (ng/mL)
Q05	Bear_01	0,1	Baja
Q05	Bear_04	1,43	Baja
Q06	Bear_02	0,8	Baja
Q06	Bear_03	1,803	**Baja**
Q07	Bear_05	5,74	Alta
Q07	Bear_06	6,46	Alta
Q07	Bear_11	18,08	Alta
Q07	Bear_13	19,31	Alta
Q07	Bear_14	17,88	Alta
Q07	Bear_42	0,109	Baja
Q08	Bear_07	9,76	Alta
Q08	Bear_08	8,91	Alta
Q08	Bear_09	10,35	Alta
Q08	Bear_10	12,93	**Alta**
Q08	Bear_15	26,59	Alta
Q08	Bear_16	30	Alta
Q09	Bear_19	3,69	Baja
Q09	Bear_21	9,65	Alta
Q09	Bear_23	12,16	Alta
Q09	Bear_24	14,63	Alta
Q09	Bear_25	15,79	Alta
Q09	Bear_43	7,85	Alta
Q10	Bear_17	2,28	Baja
Q10	Bear_18	2,69	Baja
Q10	Bear_20	6,45	Alta
Q10	Bear_22	8,88	Alta
Q10	Bear_26	15,61	Alta
Q10	Bear_27	16,4	Alta
Q10	Bear_29	16,11	Alta
Q10	Bear_30	13,52	Alta
Q10	Bear_31	18,94	Alta
Q10	Bear_32	17,12	Alta
Q11	Bear_36	0,882	Baja
Q11	Bear_37	1,382	Baja
Q11	Bear_38	1,487	Baja
Q11	Bear_39	0,669	Baja
Q11	Bear_40	0,812	Baja
Q12	Bear_34	0,191	Baja
Q12	Bear_35	0,186	Baja
Q12	Bear_41	3,61	Baja
Q13	Bear_44	0,601	**Baja**
