# Supplementary material for: Analysis of seminal plasma from brown bear (Ursus arctos) during the breeding season: Its relationship with testosterone levels
Source: PLoS One. 2017 Aug 3;12(8):e0181776. doi: 10.1371/journal.pone.0181776 (PMC5542667; doi:10.1371/journal.pone.0181776)
Supplement: S1 File — A file included with the name “RAW Data_Anel Lopez 2017.zip” have all the extra data from where we obtained the data presented in this work as follow: Data A; This is the global testosterone curve_data. Data B This is the testosterone level//bears 01–44. Data C; These are the seminal plasma gels//Bears 01–48. Data D; This is the biochemical data. Data E; This is the Zn level. Data F; Gels INBIOTEC assessment. Data G; Sperm basic and CASA parameters_database. (ZIP) [file pone.0181776.s001.zip › RAW Data_Anel Lopez 2017_REVISED/Data C.pdf]

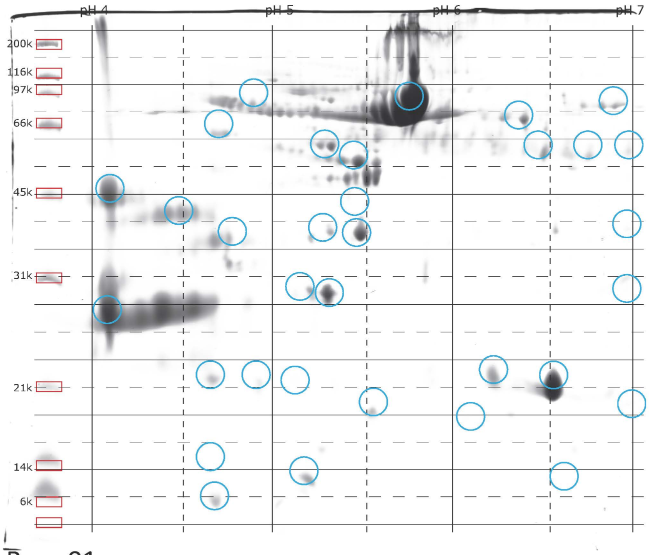

Bear 01

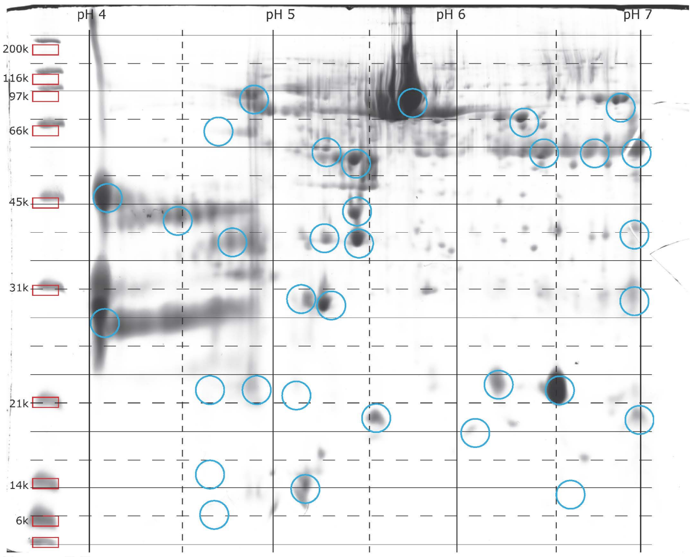

Bear 02

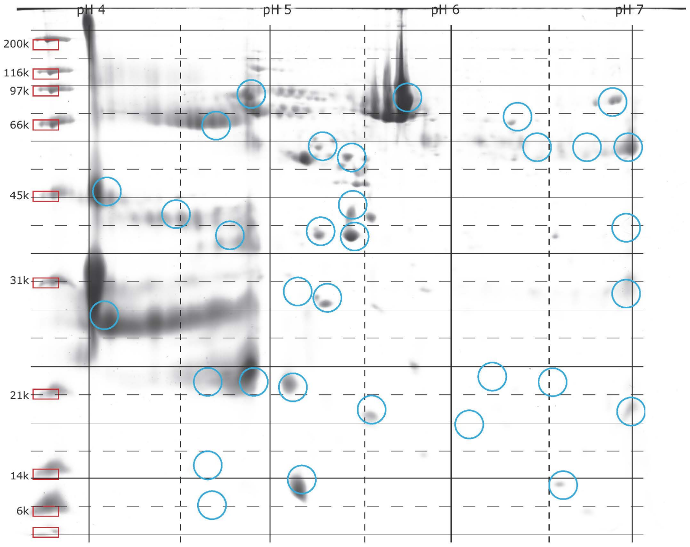

Bear 03

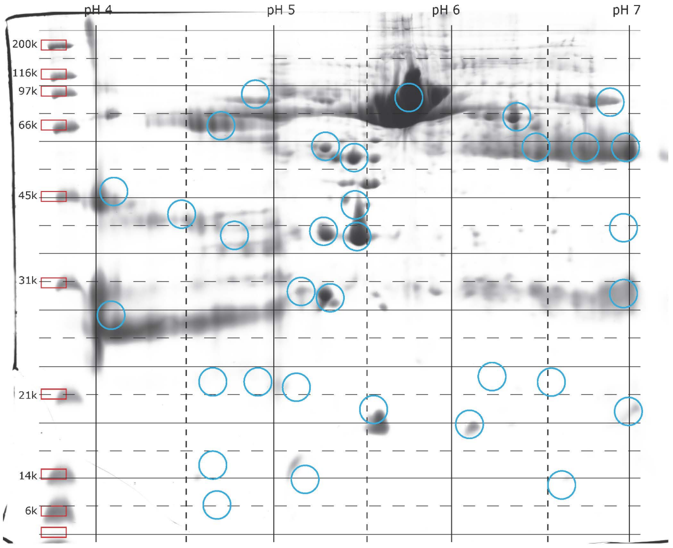

Bear 04

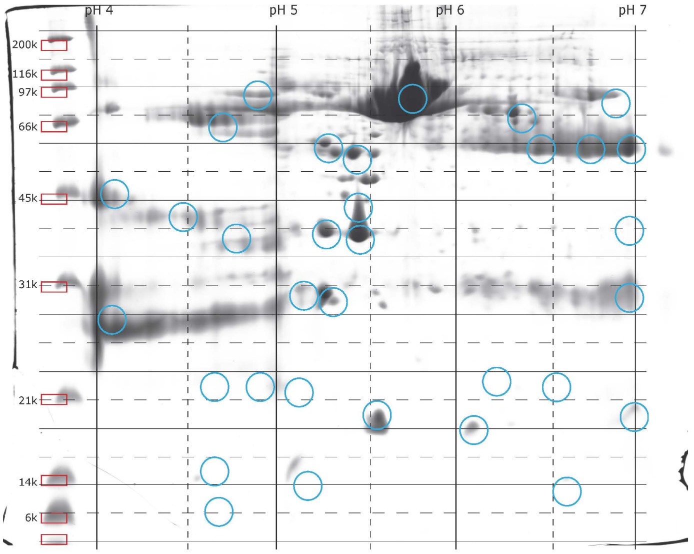

Bear 05

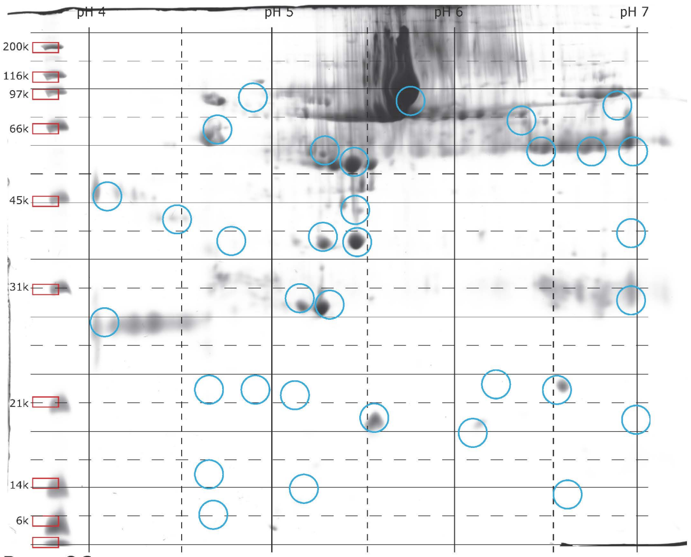

Bear 06

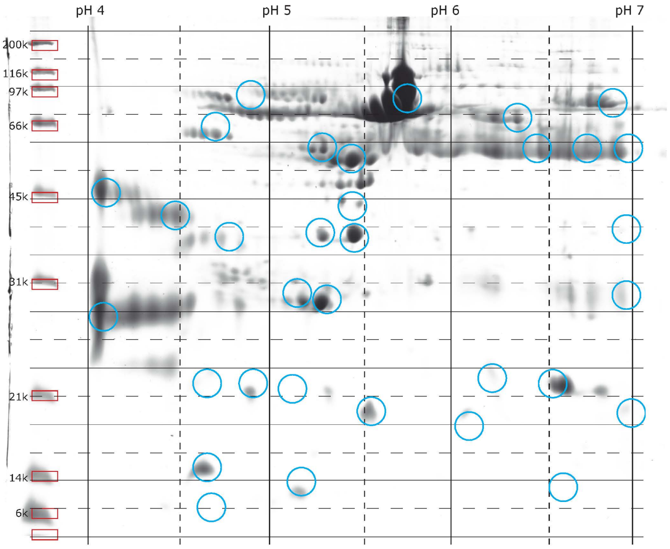

Bear 07

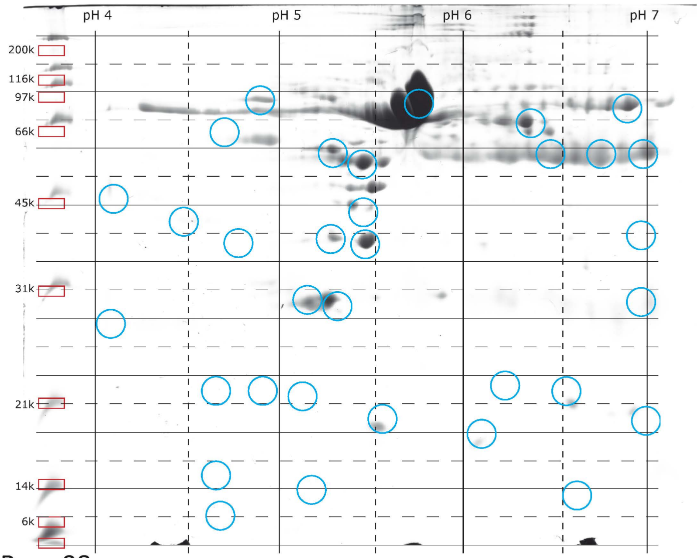

Bear 08

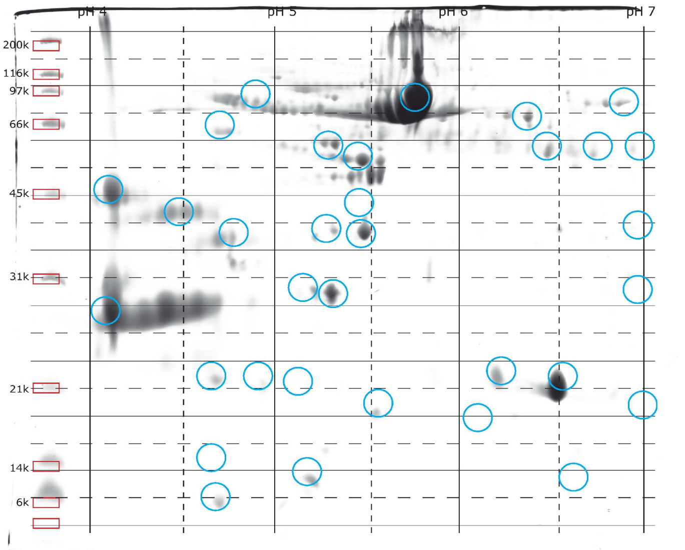

Bear 09

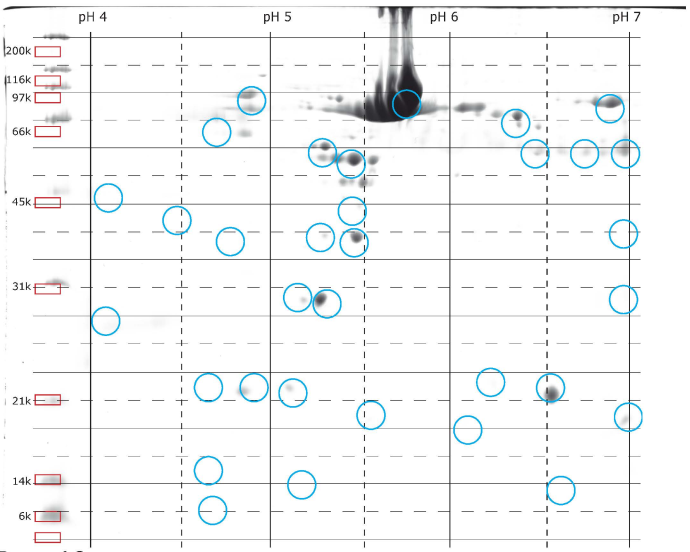

Bear 10

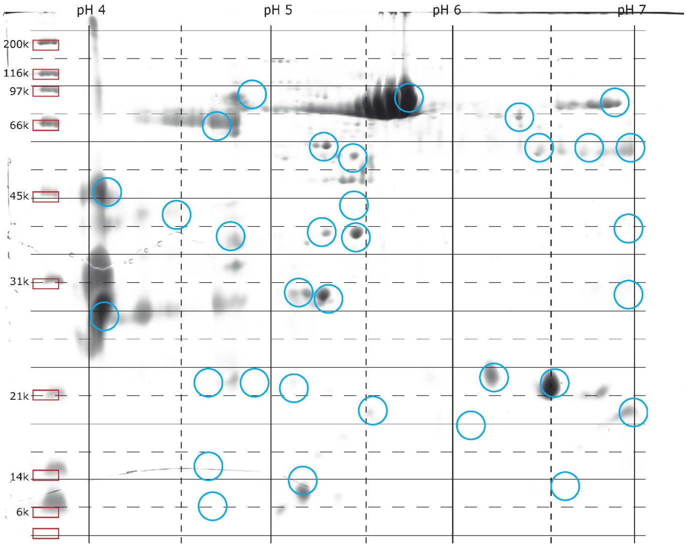

Bear 11

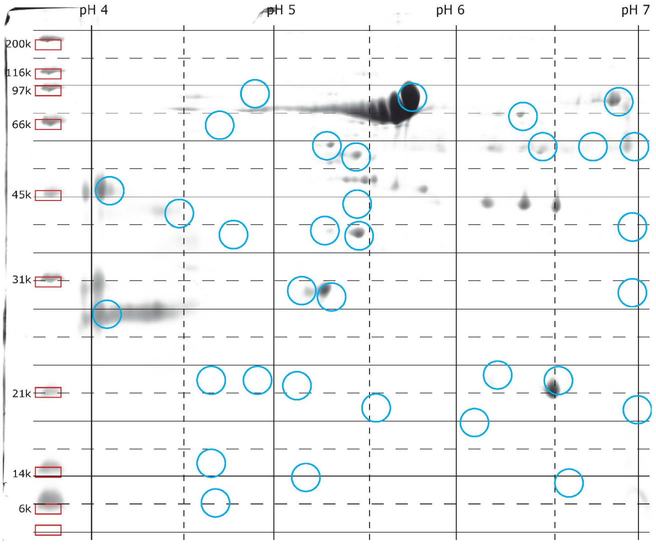

Bear 12

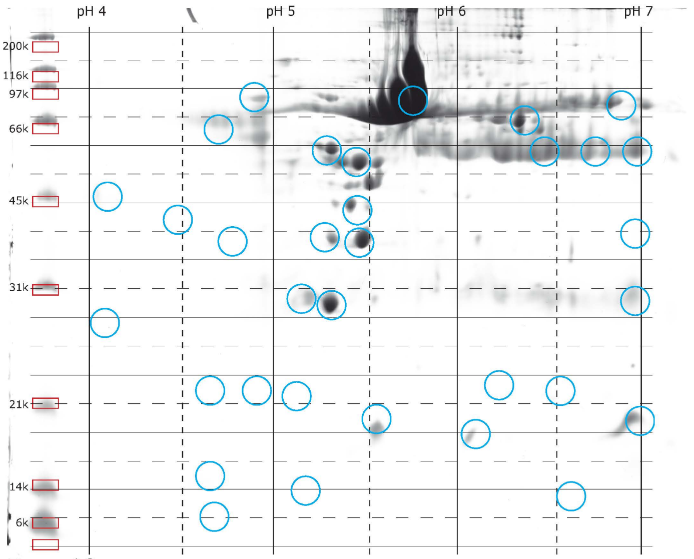

Bear 13

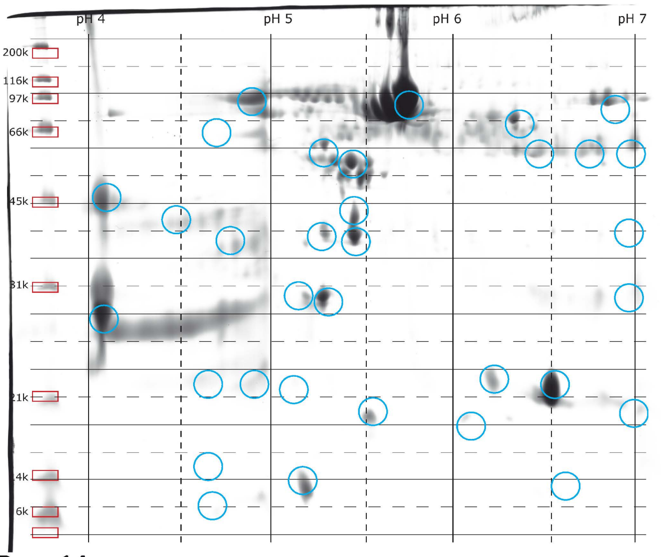

Bear 14

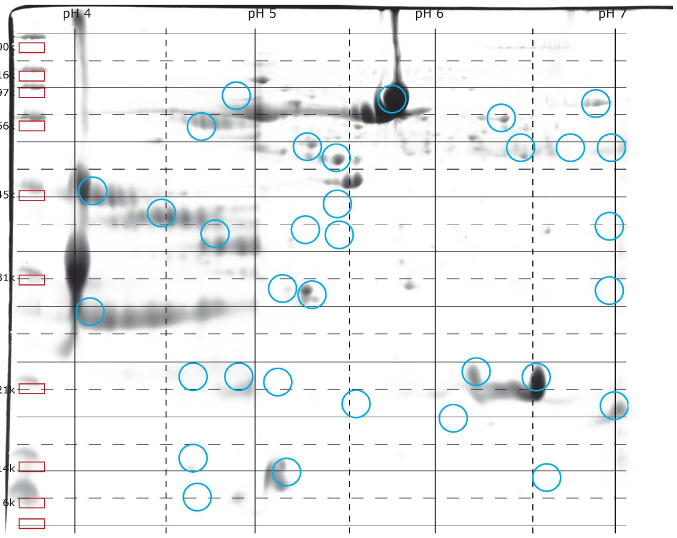

Bear 15

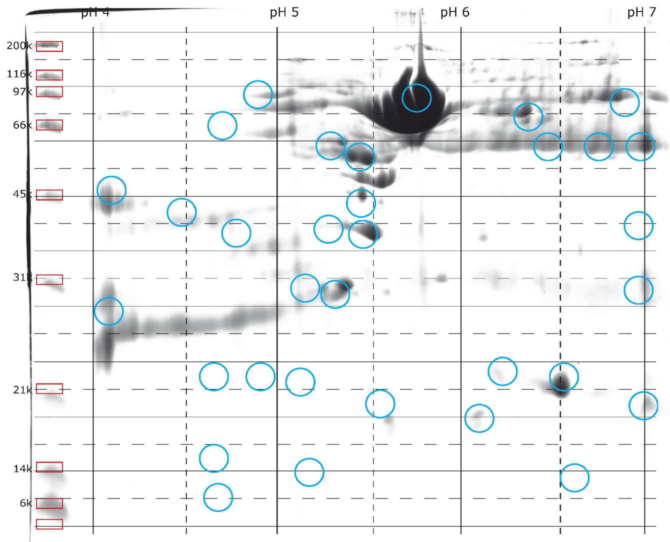

Bear 16

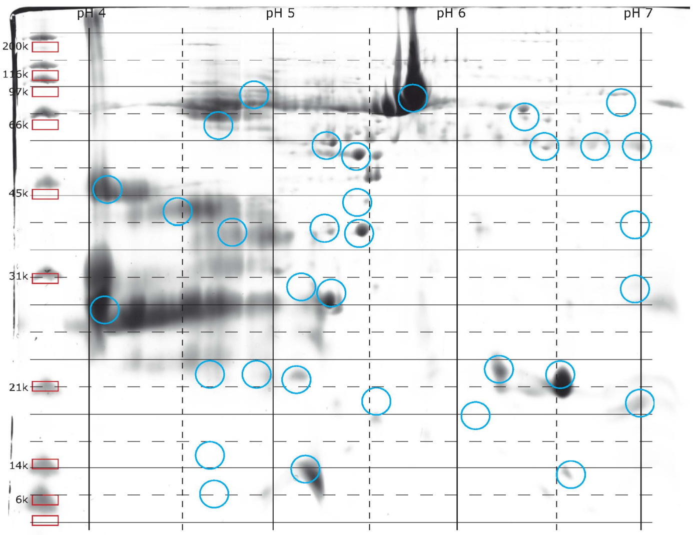

Bear 17

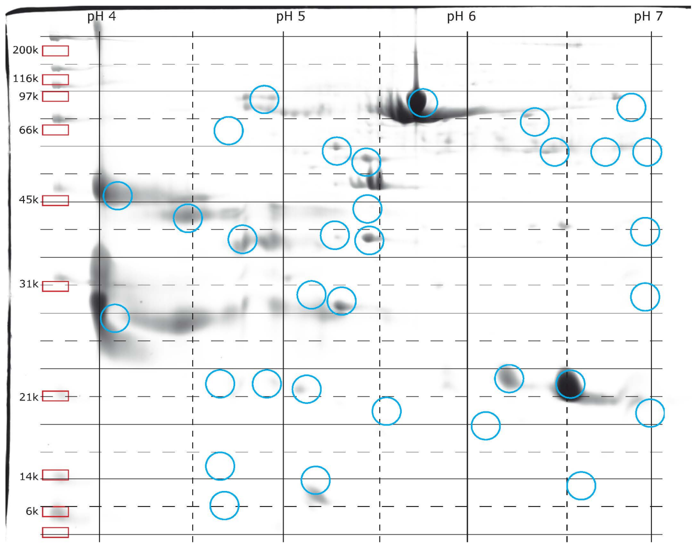

Bear 18

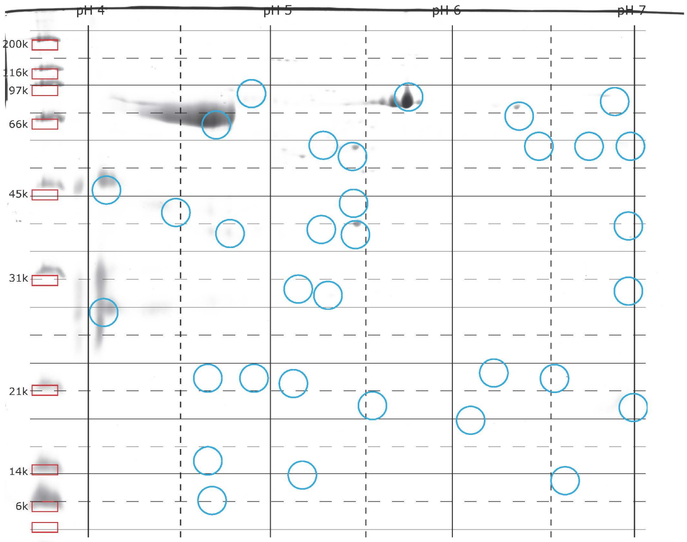

Bear 19

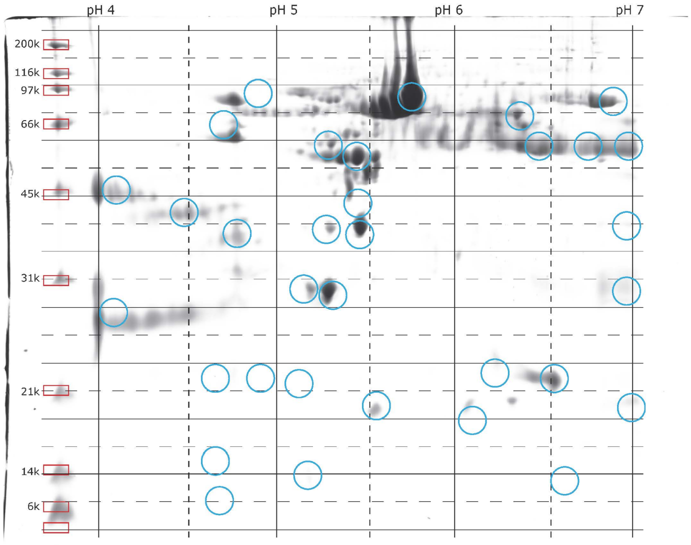

Bear 20

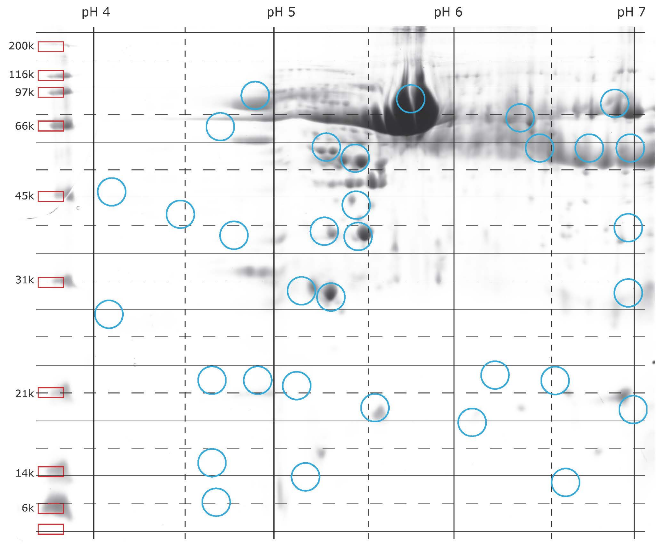

Bear 21

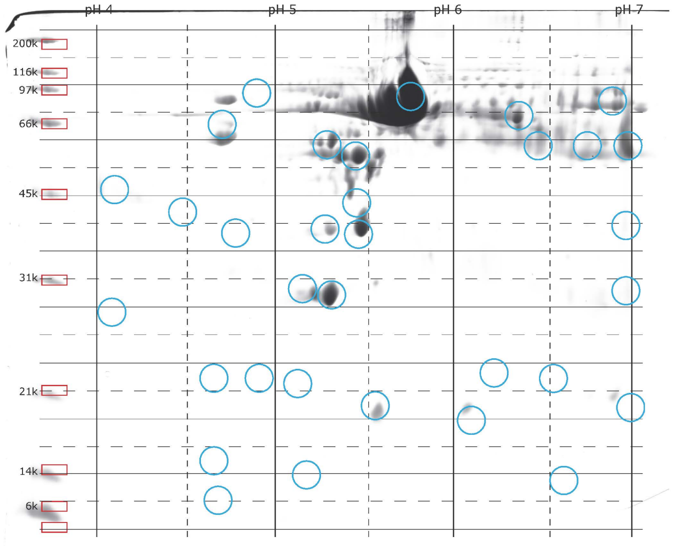

Bear 22

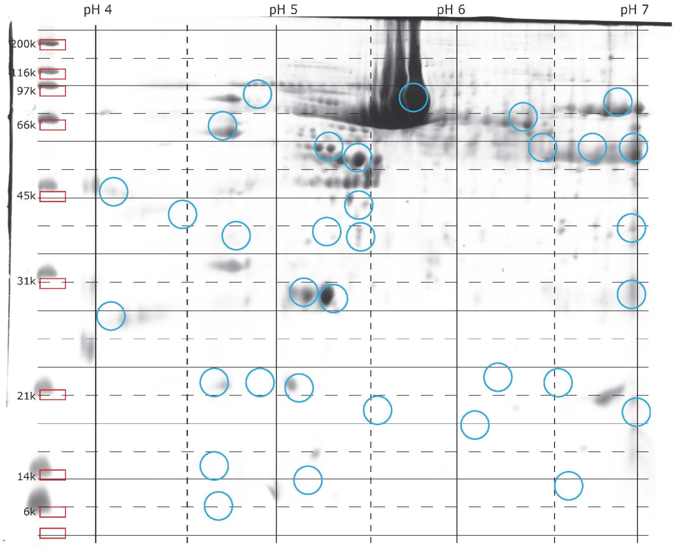

Bear 23

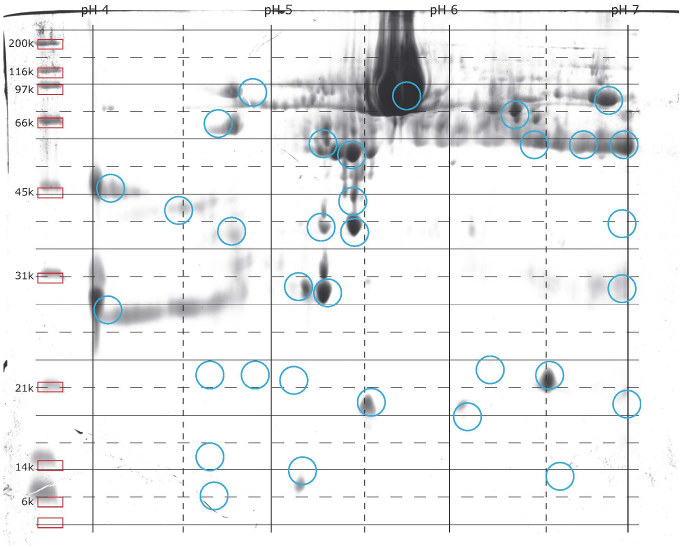

Bear 24

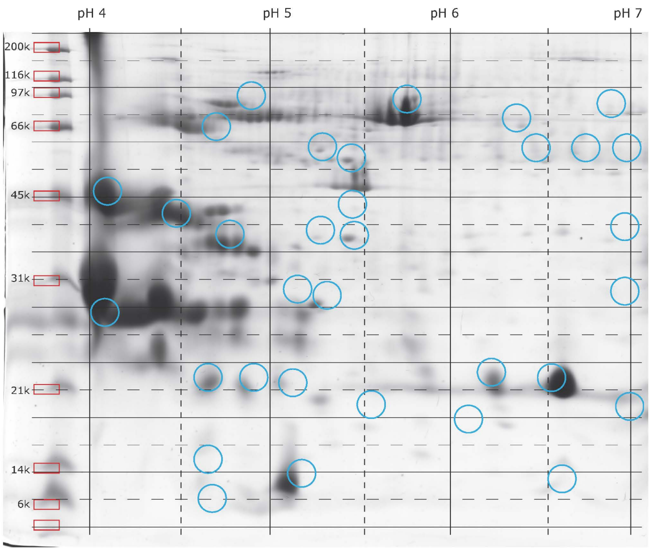

Bear 25

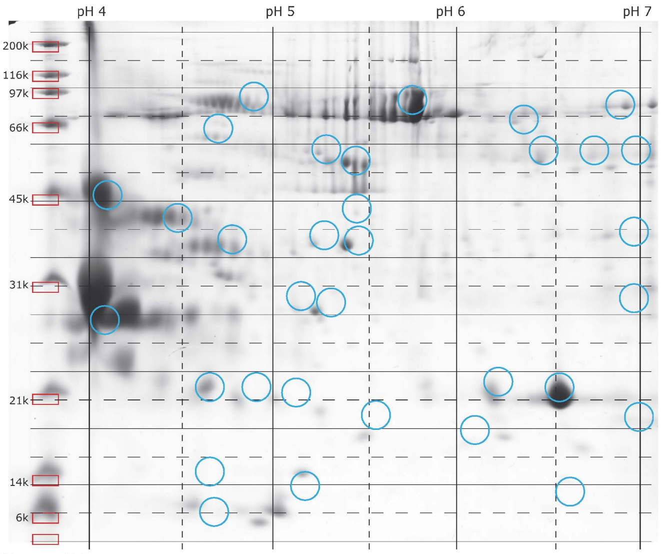

Bear 26

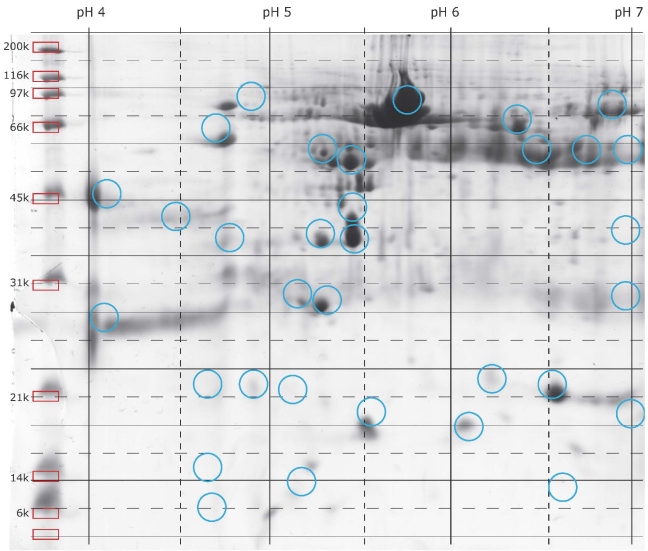

Bear 27

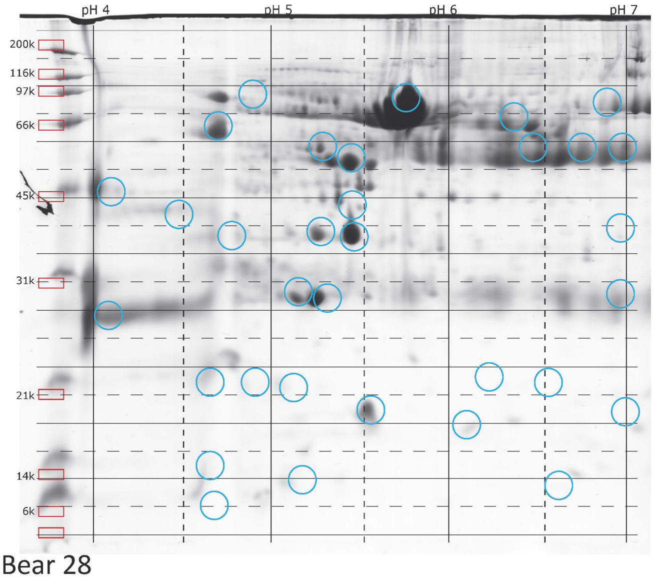

Bear 28

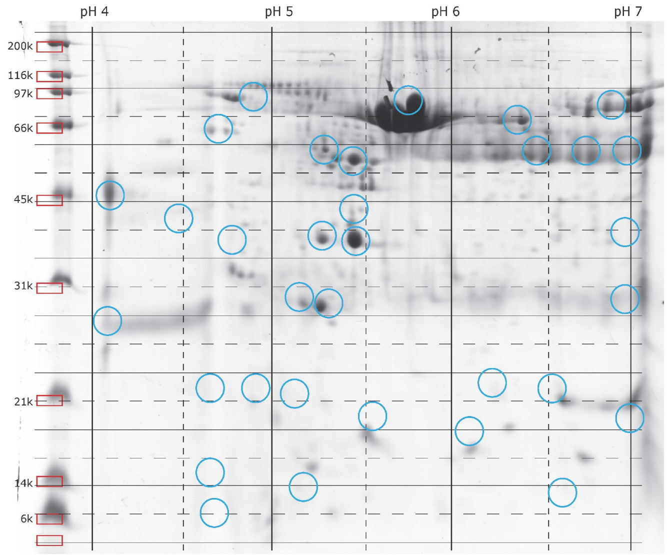

Bear 29

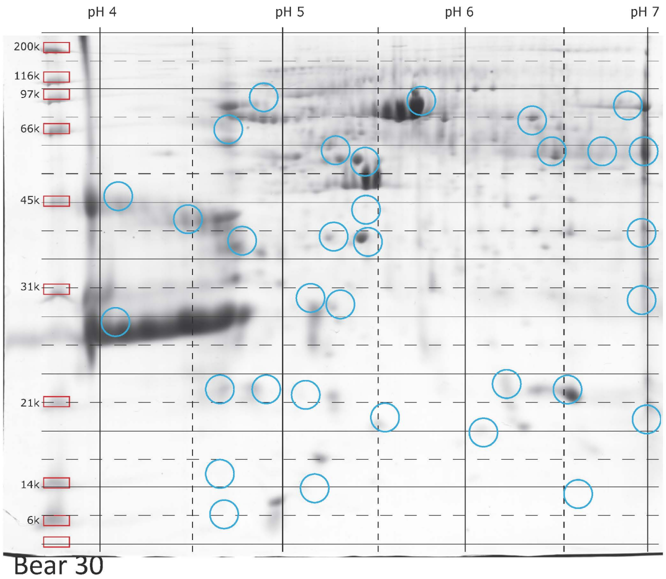

Bear 30

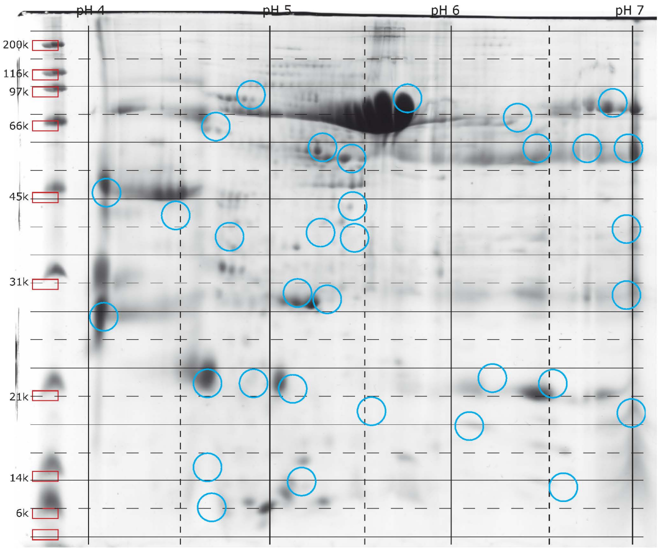

Bear 31

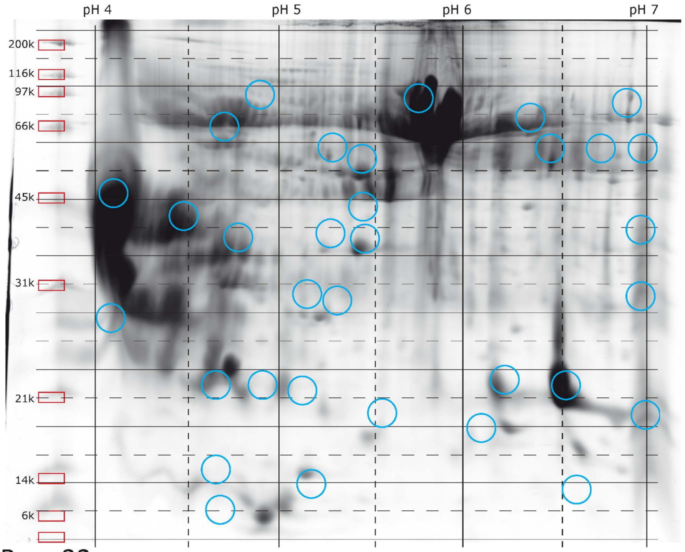

Bear 32

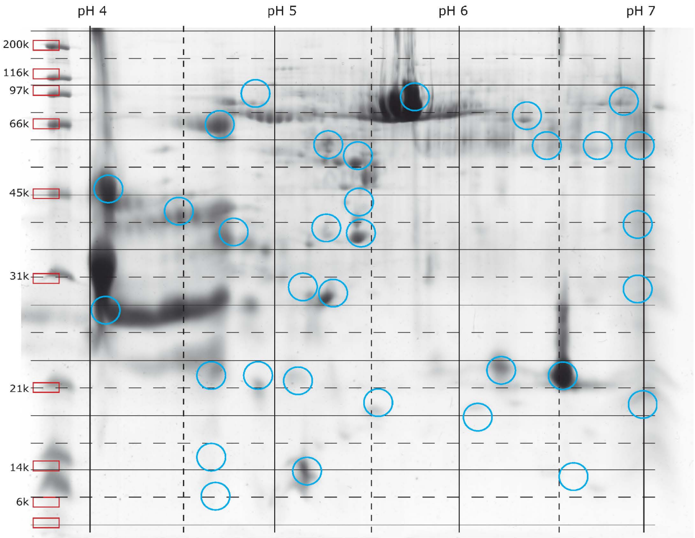

Bear 33

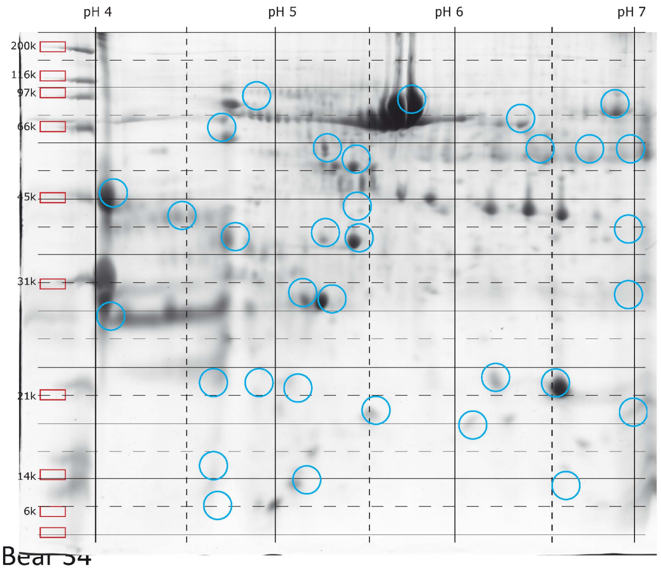

Bear 34

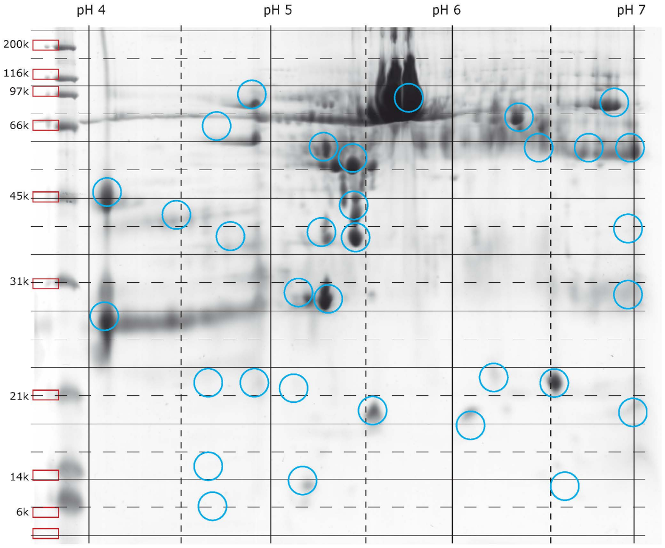

Bear 35

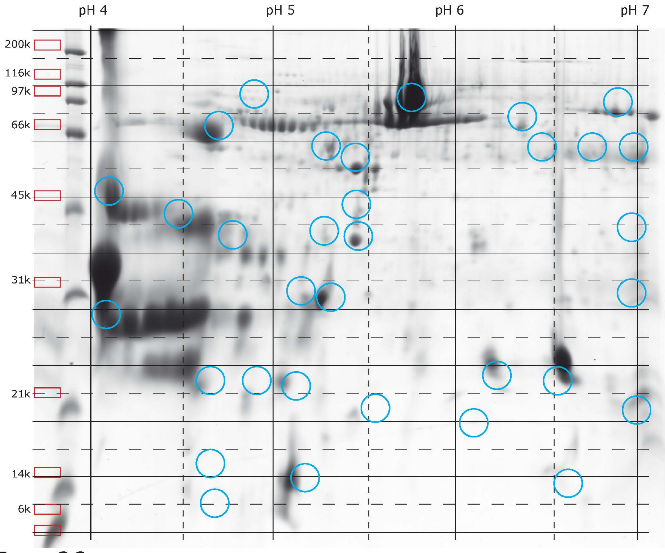

Bear 36

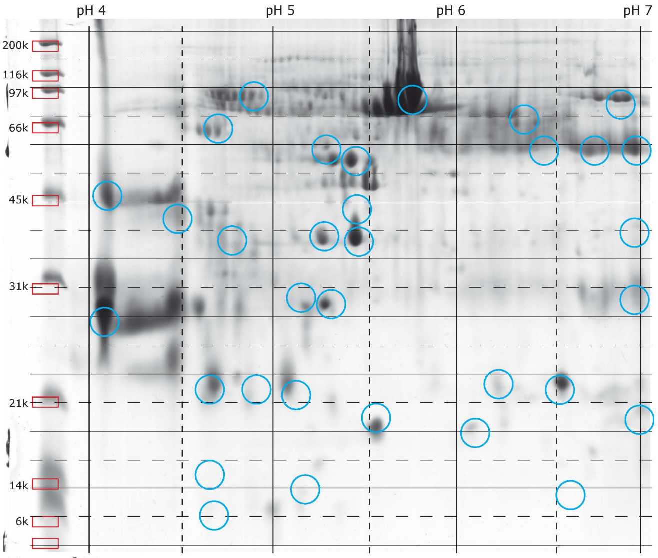

Bear 37

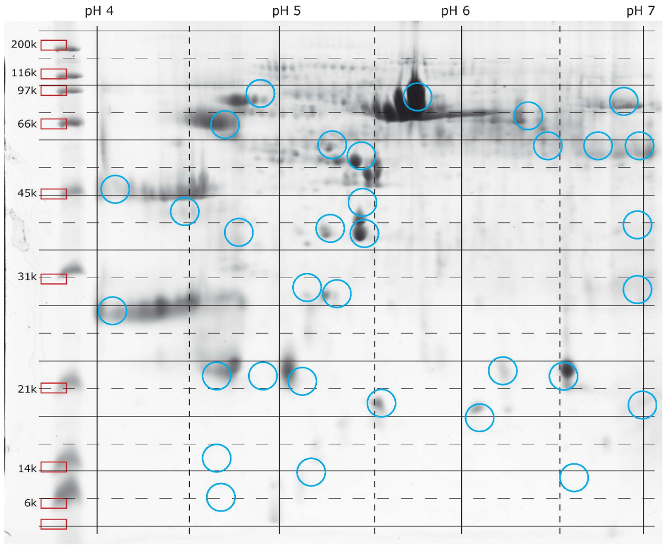

Bear 38

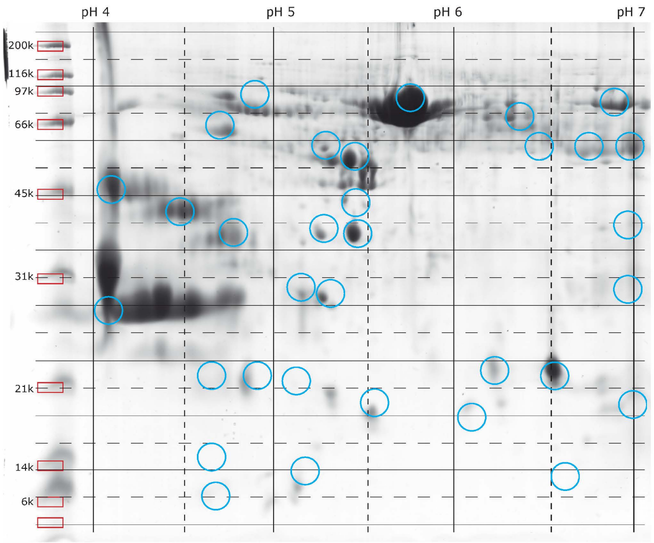

Bear 39

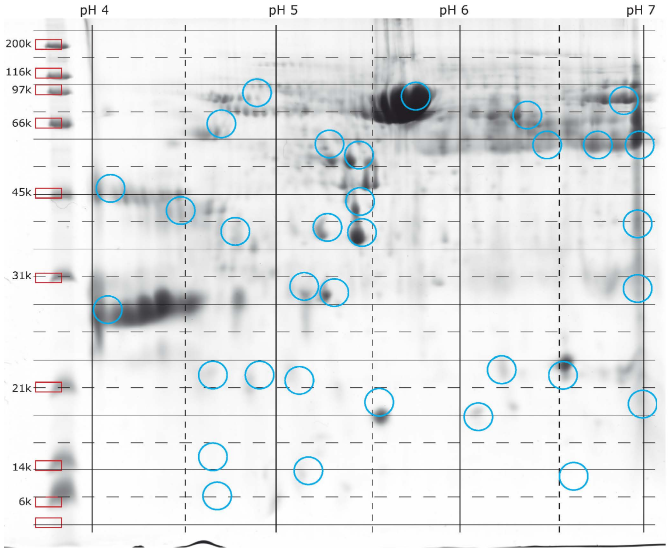

Bear 40

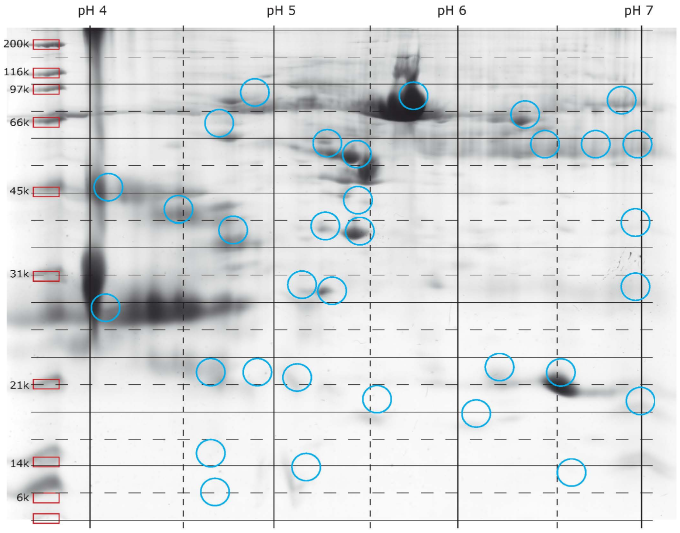

Bear 41

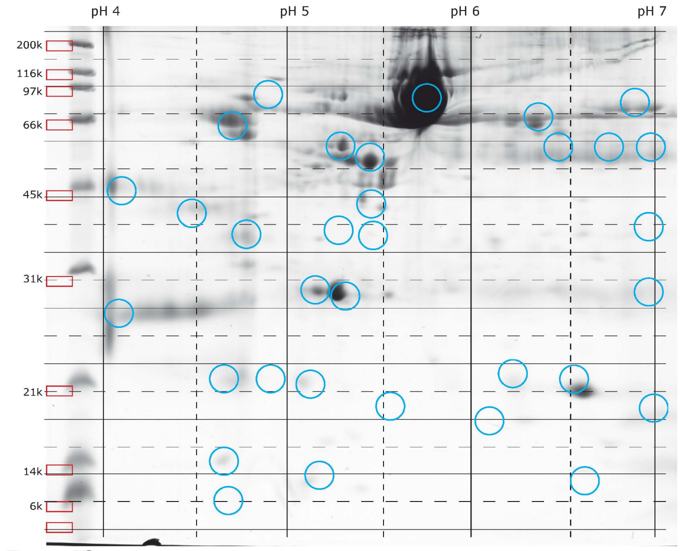

Bear 42

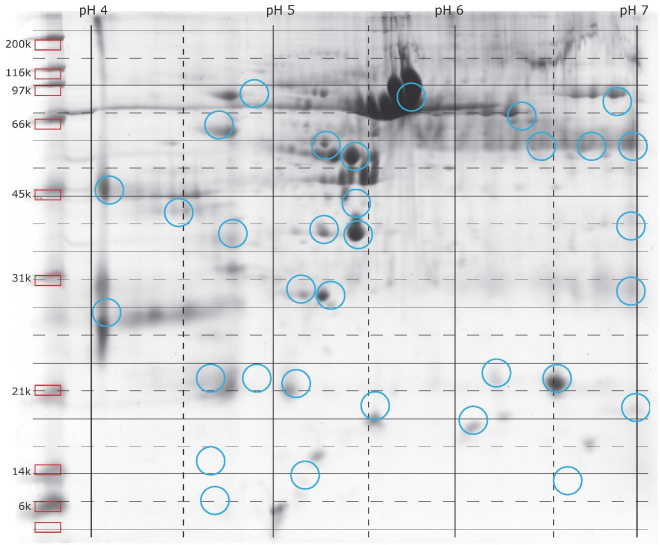

Bear 43

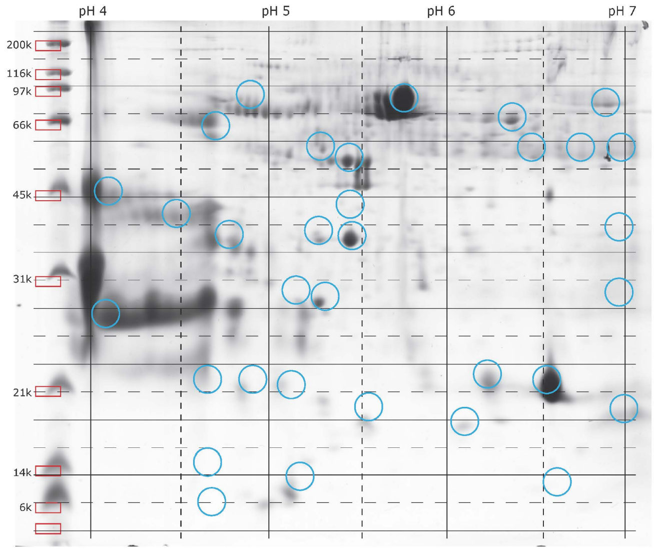

Bear 44

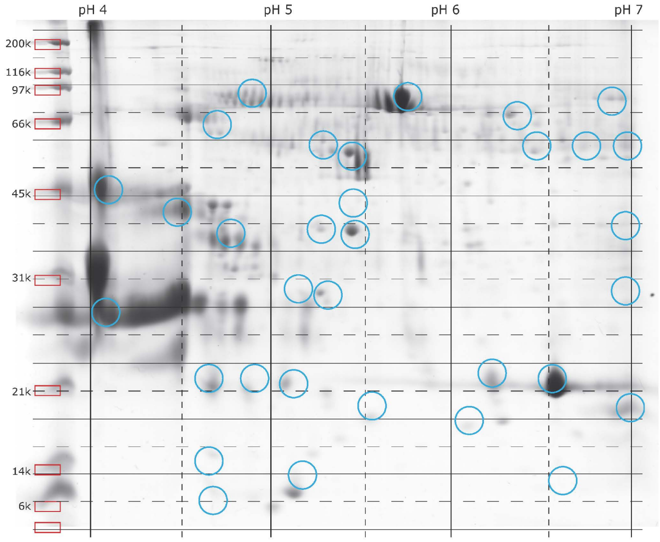

Bear 45

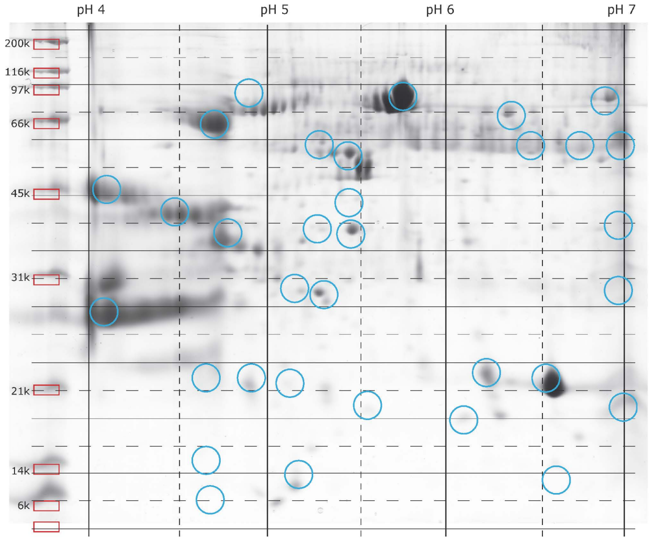

Bear 46

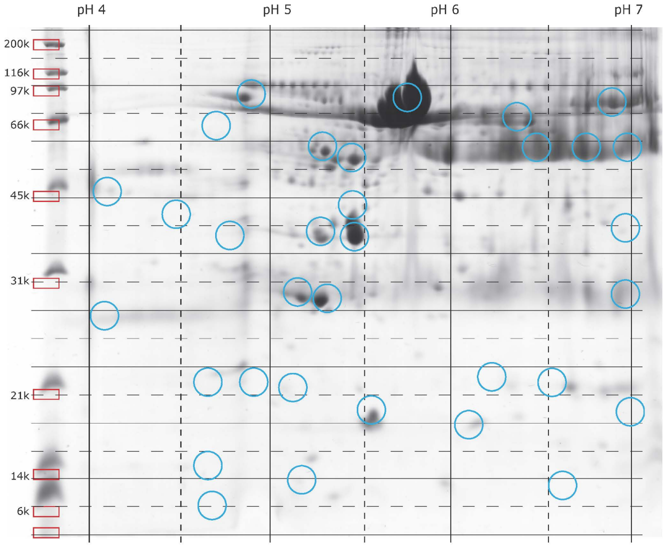

Bear 47

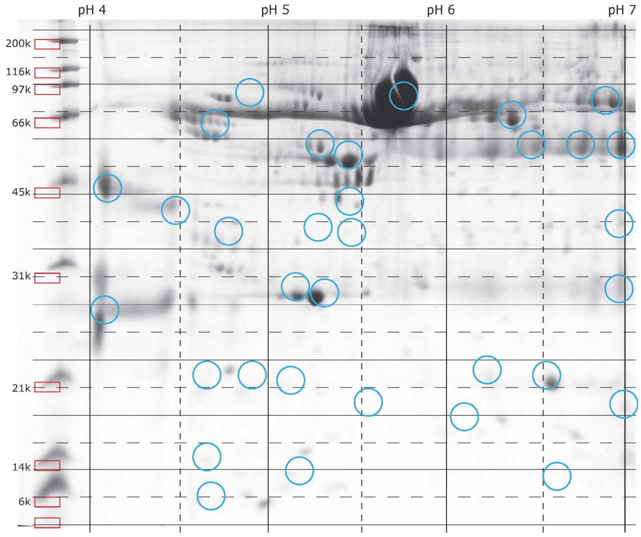

Bear 48

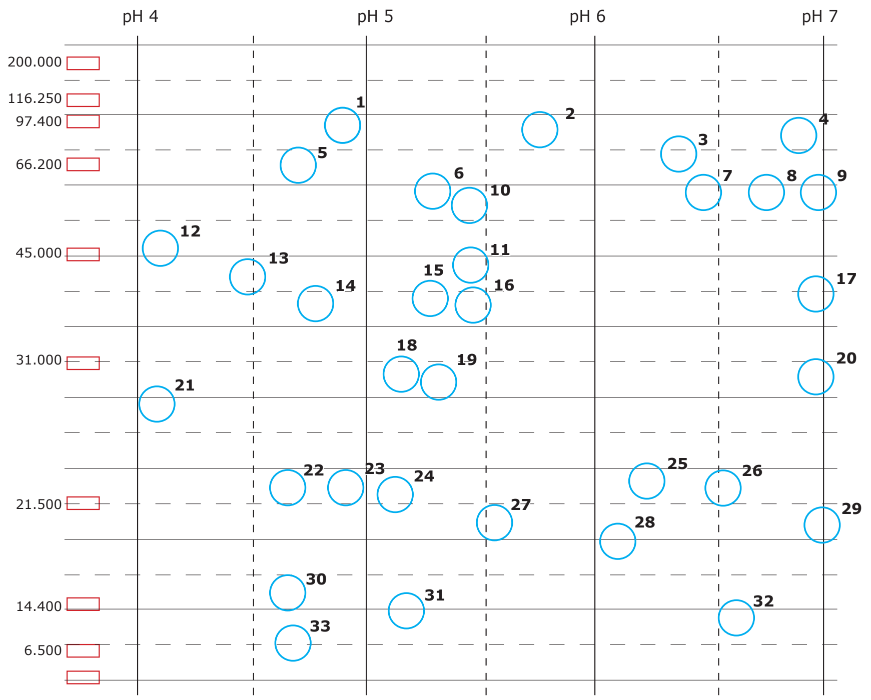
pH 4
pH 5
pH 6
pH 7
200.000
116.250
97.400
66.200
45.000
31.000
21.500
14.400
6.500
1
2
3
4
5
6
7
8
9
10
11
12
13
14
15
16
17
18
19
20
21
22
23
24
25
26
27
28
29
30
31
32
33
