# Supplementary material for: Analysis of seminal plasma from brown bear (Ursus arctos) during the breeding season: Its relationship with testosterone levels
Source: PLoS One. 2017 Aug 3;12(8):e0181776. doi: 10.1371/journal.pone.0181776 (PMC5542667; doi:10.1371/journal.pone.0181776)
Supplement: S1 File — A file included with the name “RAW Data_Anel Lopez 2017.zip” have all the extra data from where we obtained the data presented in this work as follow: Data A; This is the global testosterone curve_data. Data B This is the testosterone level//bears 01–44. Data C; These are the seminal plasma gels//Bears 01–48. Data D; This is the biochemical data. Data E; This is the Zn level. Data F; Gels INBIOTEC assessment. Data G; Sperm basic and CASA parameters_database. (ZIP) [file pone.0181776.s001.zip › RAW Data_Anel Lopez 2017_REVISED/Data E.pdf]

Muestra	Testosterona	ZINC	Unidades_ZINC
S01	Baja	16,2	µg/dL
S02	Baja	19,3	µg/dL
S03	Baja	35,0	µg/dL
S04	Baja		µg/dL
S05	Baja		µg/dL
S06	Baja	36,1	µg/dL
S07	Baja	23,5	µg/dL
S08	Baja	30,8	µg/dL
S09	Baja	23,0	µg/dL
S10	Baja		µg/dL
S11	Alta		µg/dL
S12	Alta	15,1	µg/dL
S13	Alta	27,2	µg/dL
S14	Alta	20,6	µg/dL
S15	Alta		µg/dL
S16	Alta	41,3	µg/dL
S17	Alta	21,6	µg/dL
S18	Alta	27,5	µg/dL
S19	Alta	23,0	µg/dL
S20	Alta	49,0	µg/dL
