# Supplementary material for: Analysis of seminal plasma from brown bear (Ursus arctos) during the breeding season: Its relationship with testosterone levels
Source: PLoS One. 2017 Aug 3;12(8):e0181776. doi: 10.1371/journal.pone.0181776 (PMC5542667; doi:10.1371/journal.pone.0181776)
Supplement: S1 File — A file included with the name “RAW Data_Anel Lopez 2017.zip” have all the extra data from where we obtained the data presented in this work as follow: Data A; This is the global testosterone curve_data. Data B This is the testosterone level//bears 01–44. Data C; These are the seminal plasma gels//Bears 01–48. Data D; This is the biochemical data. Data E; This is the Zn level. Data F; Gels INBIOTEC assessment. Data G; Sperm basic and CASA parameters_database. (ZIP) [file pone.0181776.s001.zip › RAW Data_Anel Lopez 2017_REVISED/Data F.pdf]

ID	Testosterone	Gel	SpotGel	X	Y	pI	MW	Vol	Area
A-14	alta	44---10-06-11	1324	843	218	6,995	76816	0,233	3,060
A-14	alta	45---10-06-11	1215	846	209	7,071	77053	0,125	2,472
A-14	alta	48---10-06-11	1449	846	221	7,071	77053	0,441	2,794
A-14	alta	15---10-06-01	1096	884	215	7,030	77053	0,159	2,710
A-14	baja	26---10-06-07	1149	826	225	7,040	77053	0,048	1,395
A-14	baja	29---10-06-07	1357	825	196	7,061	77053	0,368	3,935
A-14	baja	30---10-06-07	1156	833	203	7,050	77053	0,146	2,980
A-14	baja	36---10-06-10	1198	845	197	6,990	77291	0,041	1,153
A-14	baja	46---10-06-11	1165	841	213	7,030	77291	0,121	2,290
A-14	baja	07---10-05-28	1358	848	203	7,020	77529	0,438	3,690
A-14	baja	09---10-06-01	1084	867	196	7,071	77529	0,196	2,742
A-14	baja	14---10-06-01	936	836	202	7,030	77529	0,193	2,798
A-14	baja	17---10-06-01	1465	877	190	7,040	77769	0,214	3,577
A-14	baja	20---10-06-01	1031	847	180	7,050	77769	0,565	3,484
A-15	alta	28---10-06-07	2255	888	232	7,595	71396	0,123	1,335
A-15	alta	31---10-06-07	1508	891	244	7,646	71798	0,057	2,536
A-15	alta	34---10-06-10	1290	899	239	7,636	72445	0,181	3,798
A-15	alta	37---10-06-10	1427	875	233	7,656	72445	0,223	3,407
A-15	alta	38---10-06-10	1162	869	225	7,636	72669	0,153	2,718
A-15	alta	40---10-06-10	1205	878	233	7,626	72669	0,185	2,714
A-15	alta	44---10-06-11	1348	900	237	7,575	72669	0,068	1,560
A-15	alta	45---10-06-11	1248	898	230	7,606	72669	0,052	2,399
A-15	alta	48---10-06-11	1473	895	244	7,565	72669	0,122	1,476
A-15	alta	15---10-06-01	1133	953	243	7,727	72893	0,063	1,810
A-15	baja	26---10-06-07	1166	889	247	7,686	73118	0,059	2,335
A-15	baja	29---10-06-07	1379	876	215	7,575	73118	0,140	1,827
A-15	baja	30---10-06-07	1202	890	226	7,626	73118	0,153	2,879
A-15	baja	36---10-06-10	1223	905	214	7,585	73118	0,033	1,181
A-15	baja	46---10-06-11	1191	896	237	7,606	73118	0,052	1,512
A-15	baja	07---10-05-28	1395	900	223	7,545	73118	0,078	1,548
A-15	baja	09---10-06-01	1105	913	218	7,535	73344	0,090	1,851
A-15	baja	14---10-06-01	963	886	224	7,545	73344	0,142	2,520
A-15	baja	17---10-06-01	1507	927	212	7,545	73344	0,066	1,734
A-15	baja	20---10-06-01	1072	892	204	7,525	73344	0,108	0,931
A-16	alta	28---10-06-07	2271	139	242	4,071	64710	0,055	2,278
A-16	alta	31---10-06-07	1532	150	265	4,121	65995	0,082	2,266
A-16	alta	34---10-06-10	1339	147	278	4,107	65995	0,077	1,702
A-16	alta	37---10-06-10	1494	139	275	3,991	66741	0,058	2,492
A-16	alta	44---10-06-11	1381	132	270	3,893	67306	0,100	3,694
A-16	alta	45---10-06-11	1305	167	273	4,148	67685	0,126	4,165
A-16	alta	15---10-06-01	1157	220	260	4,319	68066	0,131	1,883
A-16	baja	29---10-06-07	1411	140	238	4,135	68258	0,092	1,601
A-16	baja	14---10-06-01	1004	161	263	4,189	70400	0,018	1,117
A-17	alta	28---10-06-07	2285	359	253	5,254	62565	0,079	1,923
A-19	alta	31---10-06-07	1610	472	313	5,564	59147	0,029	0,500
A-19	alta	34---10-06-10	1393	479	325	5,567	59816	0,070	1,085
A-19	alta	38---10-06-10	1268	476	295	5,558	60491	0,118	0,726
A-19	alta	44---10-06-11	1442	503	312	5,567	60662	0,067	0,879
A-19	alta	45---10-06-11	1344	497	299	5,555	60662	0,043	0,835
A-19	alta	28---10-06-07	1628	451	302	5,412	60584	0,067	0,703
A-19	baja	30---10-06-07	1355	501	306	5,365	61083	0,059	0,875
A-19	baja	07---10-05-28	1090	423	294	5,537	59843	0,069	0,901
A-19	baja	46---10-06-11	1285	497	309	5,555	61003	0,066	1,085
A-23	alta	28---10-06-07	2400	504	368	5,680	48382	0,376	2,843
A-23	alta	31---10-06-07	1679	511	387	5,677	49138	0,053	2,004

A-23	alta	34---10-06-10	1454	516	392	5,677	49138	0,088	2,125
A-23	alta	37---10-06-10	1614	522	386	5,697	49276	0,117	1,879
A-23	alta	38---10-06-10	1347	521	369	5,706	49554	0,085	2,593
A-23	alta	40---10-06-10	1390	529	390	5,714	49693	0,212	2,065
A-23	alta	44---10-06-11	1513	543	393	5,700	50114	0,028	2,327
A-23	alta	45---10-06-11	1422	536	381	5,685	50538	0,020	1,476
A-23	alta	48---10-06-11	1623	522	394	5,677	50538	0,329	2,290
A-23	alta	15---10-06-01	1281	571	377	5,685	50965	0,027	1,891
A-23	baja	29---10-06-07	1577	499	363	5,677	51541	0,038	1,548
A-23	baja	46---10-06-11	1354	540	389	5,688	51686	0,055	1,673
A-23	baja	07---10-05-28	1571	529	376	5,674	51832	0,196	1,778
A-23	baja	09---10-06-01	1226	546	351	5,682	52861	0,045	0,815
A-23	baja	17---10-06-01	1684	564	368	5,688	53911	0,022	1,008
A-26	alta	28---10-06-07	2461	307	445	5,104	39638	0,144	3,940
A-26	alta	31---10-06-07	1761	301	493	5,069	39710	0,104	2,714
A-26	alta	34---10-06-10	1521	324	489	5,127	39926	0,419	6,770
A-26	alta	37---10-06-10	1673	302	486	5,046	40071	0,220	5,915
A-26	alta	38---10-06-10	1406	297	468	5,061	40363	0,498	7,540
A-26	alta	44---10-06-11	1555	326	485	5,081	40436	0,640	7,597
A-26	alta	45---10-06-11	1485	328	489	5,090	40804	0,660	5,956
A-26	alta	48---10-06-11	1689	303	505	5,058	40878	0,143	3,944
A-26	baja	26---10-06-07	1355	314	500	5,078	41026	0,440	5,169
A-26	baja	29---10-06-07	1661	293	467	5,084	41101	0,037	1,452
A-33	alta	28---10-06-07	2550	390	572	5,344	31909	0,346	4,847
A-33	alta	31---10-06-07	1850	406	609	5,373	32993	0,513	5,214
A-33	alta	34---10-06-10	1601	412	604	5,376	33159	0,190	4,500
A-33	alta	37---10-06-10	1738	409	594	5,347	33159	0,220	6,641
A-33	alta	40---10-06-10	1529	445	600	5,434	33381	0,395	6,185
A-33	alta	40---10-06-10	1535	413	598	5,347	33493	0,064	1,972
A-33	alta	44---10-06-11	1624	425	600	5,350	33493	0,038	2,048
A-33	alta	45---10-06-11	1567	428	621	5,364	33549	0,123	6,060
A-33	alta	48---10-06-11	1767	403	612	5,341	33549	0,282	3,149
A-33	alta	15---10-06-01	1397	460	568	5,370	33549	0,018	1,218
A-33	baja	26---10-06-07	1432	404	627	5,341	33549	0,023	2,206
A-33	baja	29---10-06-07	1745	388	569	5,359	33605	0,193	4,093
A-33	baja	46---10-06-11	1501	457	605	5,448	33661	0,077	2,270
A-33	baja	07---10-05-28	1735	416	591	5,356	33661	0,187	3,403
A-33	baja	09---10-06-01	1348	434	569	5,361	33717	0,056	1,895
A-33	baja	17---10-06-01	1835	468	580	5,414	34105	0,235	5,956
A-37	alta	28---10-06-07	2633	323	990	5,150	15953	0,015	2,141
A-37	alta	31---10-06-07	1993	333	1042	5,162	16006	0,219	3,359
A-37	alta	34---10-06-10	1693	343	1041	5,182	16006	0,242	8,504
A-37	alta	37---10-06-10	1838	335	1006	5,139	16060	0,028	3,734
A-37	alta	44---10-06-11	1709	350	1011	5,147	16114	0,046	5,137
A-37	alta	45---10-06-11	1669	356	1036	5,165	16303	0,070	5,383
A-37	alta	48---10-06-11	1862	331	1053	5,139	16358	0,065	2,198
A-37	baja	26---10-06-07	1517	328	1056	5,119	16522	0,155	5,472
A-37	baja	29---10-06-07	1866	323	1021	5,171	16745	0,093	3,617
A-37	baja	36---10-06-10	1624	338	906	5,136	16885	0,011	1,972
A-37	baja	46---10-06-11	1582	371	1047	5,217	16913	0,056	2,815
A-37	baja	07---10-05-28	1860	350	1015	5,174	17783	0,073	5,190
B-03	alta	28---10-06-07	2239	693	217	6,323	73571	0,082	1,290
B-03	alta	31---10-06-07	1499	690	235	6,292	73798	0,103	1,819
B-03	alta	34---10-06-10	1282	708	233	6,349	74484	0,047	1,226
B-03	alta	37---10-06-10	1417	717	218	6,478	74714	0,024	0,653
B-03	alta	38---10-06-10	1147	722	208	6,525	75641	0,052	0,734

B-03	alta	40---10-06-10	1191	721	219	6,478	75874	0,121	1,573
B-03	alta	44---10-06-11	1327	728	216	6,390	76109	0,091	1,371
B-03	alta	45---10-06-11	1230	721	210	6,370	76109	0,058	1,286
B-03	alta	48---10-06-11	1403	700	210	6,282	76109	0,440	2,157
B-03	alta	15---10-06-01	1100	767	216	6,406	76109	0,091	1,238
B-03	baja	26---10-06-07	1153	708	228	6,396	76579	0,044	0,577
B-03	baja	29---10-06-07	1376	703	209	6,406	76579	0,146	1,173
B-03	baja	30---10-06-07	1182	707	209	6,370	76579	0,190	1,536
B-03	baja	36---10-06-10	1212	745	207	6,468	77053	0,053	1,141
B-03	baja	46---10-06-11	1164	722	212	6,370	77291	0,060	1,020
B-03	baja	07---10-05-28	1364	714	202	6,323	77529	0,100	1,351
B-03	baja	09---10-06-01	1093	733	206	6,349	77529	0,073	0,738
B-03	baja	14---10-06-01	946	712	208	6,365	77769	0,033	0,645
B-03	baja	17---10-06-01	1487	750	191	6,359	77769	0,036	0,407
B-03	baja	20---10-06-01	1020	727	179	6,385	80452	0,320	3,371
B-06	alta	31---10-06-07	1617	519	319	5,700	57671	0,072	2,024
B-06	alta	15---10-06-01	1227	568	316	5,677	57996	0,049	1,222
B-06	alta	14---10-06-01	1062	537	305	5,185	51917	0,061	1,652
B-06	baja	26---10-06-07	1269	510	330	5,659	58159	0,072	1,508
B-06	baja	29---10-06-07	1481	511	294	5,709	58159	0,141	1,984
B-06	baja	30---10-06-07	1313	515	313	5,671	58651	0,046	0,685
B-06	baja	36---10-06-10	1338	537	295	5,694	58981	0,081	1,149
B-06	baja	46---10-06-11	1301	536	322	5,677	58981	0,056	1,109
B-06	baja	07---10-05-28	1516	528	317	5,671	59147	0,076	1,488
B-06	baja	14---10-06-01	1052	527	315	5,685	59147	0,198	3,052
B-06	baja	17---10-06-01	1633	562	310	5,682	59314	0,060	1,206
B-06	baja	20---10-06-01	1163	537	297	5,671	59648	0,119	1,645
B-08	alta	28---10-06-07	2526	462	536	5,555	34527	0,047	2,714
B-08	alta	31---10-06-07	1828	483	583	5,596	34740	0,061	2,548
B-08	alta	40---10-06-10	1510	489	563	5,555	35008	0,043	1,343
B-08	alta	45---10-06-11	1536	490	547	5,535	35332	0,025	0,565
B-08	alta	48---10-06-11	1733	489	560	5,584	35769	0,031	2,875
B-08	baja	29---10-06-07	1738	475	562	5,607	35825	0,038	1,161
B-08	baja	36---10-06-10	1495	501	498	5,596	35880	0,251	3,161
B-08	baja	46---10-06-11	1493	516	586	5,613	36157	0,058	1,798
B-08	baja	07---10-05-28	1710	500	561	5,587	36380	0,131	2,347
B-08	baja	17---10-06-01	1805	540	533	5,610	36548	0,044	1,238
