# Supplementary material for: Analysis of seminal plasma from brown bear (Ursus arctos) during the breeding season: Its relationship with testosterone levels
Source: PLoS One. 2017 Aug 3;12(8):e0181776. doi: 10.1371/journal.pone.0181776 (PMC5542667; doi:10.1371/journal.pone.0181776)
Supplement: S1 File — A file included with the name “RAW Data_Anel Lopez 2017.zip” have all the extra data from where we obtained the data presented in this work as follow: Data A; This is the global testosterone curve_data. Data B This is the testosterone level//bears 01–44. Data C; These are the seminal plasma gels//Bears 01–48. Data D; This is the biochemical data. Data E; This is the Zn level. Data F; Gels INBIOTEC assessment. Data G; Sperm basic and CASA parameters_database. (ZIP) [file pone.0181776.s001.zip › RAW Data_Anel Lopez 2017_REVISED/Data G.pdf]

Sample	Test_Level	Vol	Conc	SPZ	pH	PO	MIH	MPH	VAP	VCL	VSL	LIN	STR	WOB	ALH	BCF
S06	Alto	3,2	444,8	1423,5	7,76	245	64,86	36,49	73,21	113,67	55,39	43,71	67,82	64,22	4,29	7,82
S07	Alto	2,7	44,2	119,3	8,69	332	74,01	51,62	77,01	113,22	64,65	53,39	75,94	68,34	4,11	7,54
S08	Alto															
S09	Alto	1,8	423,2	761,8	8,20	255	80,06	40,80	77,84	109,66	51,64	44,07	63,11	69,31	4,20	6,18
S10	Alto	2,6	38,9	101,1	8,27	323	82,14	20,24	115,34	137,72	50,62	36,92	44,64	82,54	4,29	3,59
S12	Alto	1,0	34,4	34,4	8,09	235	78,61	49,71	68,71	109,09	52,94	43,78	69,20	60,89	4,48	6,48
S13	Alto															
S14	Alto	1,5	479,8	719,6	7,98	287	74,23	35,29	92,95	140,99	70,22	48,56	72,37	65,93	5,50	7,20
S15	Alto															
S16	Alto	1,4	146,4	205,0	8,24	281	92,82	70,51	115,33	165,59	91,66	53,89	76,66	69,32	5,89	8,74
S17	Alto															
S18	Alto	1,6	584,7	935,5	8,45	308	92,44	73,11	122,81	192,50	103,44	51,47	79,63	62,92	6,41	10,35
S19	Alto	3,2	37,2	119,1	7,42	208	69,77	18,60	93,27	115,30	39,08	35,41	45,03	79,03	3,81	3,06
S21	Alto	2,4	156,1	374,6	8,44	300	77,22	44,44	120,25	176,97	97,79	52,99	77,57	67,21	6,11	9,44
S22	Alto	2,3	100,4	230,9	8,28	300	80,63	38,34	106,55	167,52	79,47	47,11	72,68	64,08	6,57	7,64
S24	Alto	3,5	11,9	41,8	8,37	306	70,34	14,48	86,73	98,38	42,74	43,50	50,47	86,34	2,76	5,24
S25	Alto	5,3	50,4	266,9	8,51	305	88,79	21,23	132,65	152,15	73,43	48,67	56,57	85,93	3,98	3,76
S26	Alto	1,9	203,2	386,2	8,57	298	88,89	51,28	113,86	154,12	90,79	58,76	77,98	75,73	5,18	8,36
S27	Alto	2,5	252,6	631,5	8,51	302	91,22	43,90	108,74	150,41	81,64	52,90	72,18	73,35	5,26	7,67
S28	Alto	1,9	852,1	1619,0	8,41	320	85,20	75,34	149,66	205,45	134,15	63,42	86,70	72,12	5,85	12,03
S32	Alto															
S42	Alto	2,2	31,6	69,5	8,43	327	52,76	48,03	127,30	180,64	116,54	63,15	89,04	70,11	5,90	10,91
S43	Alto	2,0	74,6	149,2	8,39	326	58,01	44,20	98,58	139,72	87,36	56,83	80,09	69,06	4,65	7,84
S44	Alto	4,5	137,3	617,8	8,29	302	72,43	58,38	107,82	170,04	89,84	51,70	80,47	63,07	6,41	9,52
S45	Alto	2,0	29,2	58,4	8,16	269	84,24	48,37	106,60	150,19	71,11	45,58	64,63	71,96	5,45	6,64
S47	Alto	4,0	211,6	846,3	8,03	304	66,22	54,50	120,78	173,73	107,90	56,31	82,45	66,80	5,44	11,46
S01	Bajo	1,4	54,4	76,2	8,07	241	68,92	29,43	59,78	80,66	37,67	42,82	60,93	69,39	2,95	5,71
S02	Bajo	1,7	12,8	21,8	8,45	309	24,68	16,60	71,64	102,61	59,18	48,73	72,25	66,11	3,84	6,01
S03	Bajo	2,7	263,1	710,4	8,37		78,19	31,65	68,36	109,67	43,45	38,32	58,88	61,84	4,49	6,66
S04	Bajo	2,0	170,8	341,6	8,25	301	85,86	50,30	110,71	135,88	79,41	55,57	68,58	79,27	4,07	7,16
S05	Bajo	1,8	10,0	18,0	8,18	296	57,23	48,80	127,71	160,90	110,04	65,83	83,87	78,11	4,87	7,85
S11	Bajo	3,5	188,1	658,4	8,15	305	77,68	59,65	85,17	129,59	67,20	52,71	77,49	66,80	5,13	8,86
S20	Bajo	4,2	180,2	756,8	8,24	303	83,92	28,62	88,12	154,42	62,89	41,42	69,48	58,38	6,52	5,51
S23	Bajo	1,6	415,2	664,3	7,46	255	80,81	51,52	70,50	110,81	54,89	45,31	70,01	63,09	4,42	7,25
S29	Bajo	2,7	95,0	256,4	7,92	309	70,80	40,60	117,17	142,47	81,58	52,89	66,21	79,38	4,33	6,09
S30	Bajo	1,3	509,3	662,1	8,79	297	94,21	50,99	110,82	184,79	83,92	45,17	73,99	60,28	7,85	8,89
S31	Bajo					315										
S33	Bajo	4,2	16,0	67,2	8,63	311	71,07	44,63	106,36	149,91	78,41	50,46	69,37	71,74	4,16	5,62
S34	Bajo	3,0	105,5	316,6	8,48	275	91,62	48,48	125,57	172,72	90,69	52,10	71,23	73,34	5,75	7,63
S35	Bajo	3,0	64,5	193,5	8,23	272										
S36	Bajo	1,8	63,0	113,3	8,73		91,15	78,76	130,82	195,70	110,38	56,39	82,77	67,42	6,33	8,22
S37	Bajo	3,0	44,4	133,1	8,29	295	91,86	68,70	154,48	188,29	133,13	69,84	84,60	82,03	5,21	10,78
S38	Bajo	1,5	193,8	290,7	7,70	295	53,85	28,74	117,22	139,73	76,52	52,72	63,68	82,40	4,21	4,87
S39	Bajo	2,9	197,5	572,9	8,06	322	95,17	25,00	114,01	142,82	67,76	46,42	59,11	78,57	4,48	5,00
S40	Bajo	2,3	307,9	708,1	8,04	305	88,82	43,35	96,37	164,57	74,63	44,88	74,00	59,43	6,71	8,57
S41	Bajo	1,4	235,5	329,7	8,47	313										
S46	Bajo	2,4	146,7	352,1	7,99	271	90,73	67,32	93,22	141,88	77,37	51,83	77,80	64,86	5,44	8,51
